# Proteomics of intracellular *Salmonella enterica* reveals roles of *Salmonella* pathogenicity island 2 in metabolism and antioxidant defense

**DOI:** 10.1371/journal.ppat.1007741

**Published:** 2019-04-22

**Authors:** Janina Noster, Tzu-Chiao Chao, Nathalie Sander, Marc Schulte, Tatjana Reuter, Nicole Hansmeier, Michael Hensel

**Affiliations:** 1 Abt. Mikrobiologie, Universität Osnabrück, Osnabrück, Germany; 2 Institute of Environmental Change & Society, University of Regina, Regina, Canada; Stanford University School of Medicine, UNITED STATES

## Abstract

Intracellular *Salmonella enterica* serovar Typhimurium (STM) deploy the *Salmonella* Pathogenicity Island 2-encoded type III secretion system (SPI2-T3SS) for the massive remodeling of the endosomal system for host cells. This activity results in formation of an extensive interconnected tubular network of *Salmonella*-induced filaments (SIFs) connected to the *Salmonella*-containing vacuole (SCV). Such network is absent in cells infected with SPI2-T3SS-deficient mutant strains such as Δ*ssaV*. A tubular network with reduced dimensions is formed if SPI2-T3SS effector protein SseF is absent. Previous single cell live microscopy-based analyses revealed that intracellular proliferation of STM is directly correlated to the ability to transform the host cell endosomal system into a complex tubular network. This network may also abrogate host defense mechanisms such as delivery of antimicrobial effectors to the SCV. To test the role of SIFs in STM patho-metabolism, we performed quantitative comparative proteomics of STM recovered from infected murine macrophages. We infected RAW264.7 cells with STM wild type (WT), Δ*sseF* or Δ*ssaV* strains, recovered bacteria 12 h after infection and determined proteome compositions. Increased numbers of proteins characteristic for nutritional starvation were detected in STM Δ*sseF* and Δ*ssaV* compared to WT. In addition, STM Δ*ssaV*, but not Δ*sseF* showed signatures of increased exposure to stress by antimicrobial defenses, in particular reactive oxygen species, of the host cells. The proteomics analyses presented here support and extend the role of SIFs for the intracellular lifestyle of STM. We conclude that efficient manipulation of the host cell endosomal system by effector proteins of the SPI2-T3SS contributes to nutrition, as well as to resistance against antimicrobial host defense mechanisms.

## Author summary

The facultative intracellular bacterium *Salmonella enterica* has evolved sophisticated mechanisms to adapt to life inside a pathogen-containing vacuole in mammalian host cells. Intracellular *Salmonella* manipulate the host cell endosomal system resulting in formation of a complex network of tubular vesicles, termed *Salmonella*-induced filaments (SIFs). We applied quantitative proteomics to intracellular *Salmonella* in murine macrophages and compared the wild-type strain to mutant strains with aberrant SIF architecture, or no capacity for induction of SIF. We determined that those mutant strains contain higher amounts of transporters for nutrient uptake, and lower amounts of proteins for central carbon metabolism. These observations indicate response to nutrient restriction in absence of fully established SIF. In addition, the mutant strain unable to induce SIF formation showed increased amounts of proteins required for response to antimicrobial factors of the host cells. These data show that the massive remodeling of the endosomal system of host cells by intracellular *Salmonella* serves to essential needs, i.e. to enable access to nutrients for efficient proliferation of the pathogen, and to withstand hostile conditions within the pathogen-containing vacuole.

## Introduction

*Salmonella enterica* is an invasive, facultative intracellular pathogen causing frequent infection of humans and animal hosts that range from gastroenteritis to typhoid fever, a systemic infection caused by human-restricted *S*. *enterica* serovars such as Typhi. *S*. *enterica* serovar Typhimurium (STM) is causative agent of gastroenteritis in humans and causes systemic infection in susceptible mouse lines.

The intracellular lifestyle of STM has been intensively studied, and prior work revealed that this pathogen inhabits a unique membrane-bound compartment, that is referred to as *Salmonella*-containing vacuole or SCV [1]. The SCV has certain features of a late endosomal compartment. However, virulence factors of STM converted the SCV into an intracellular habitat that is permissive for intracellular survival and replication. A remarkable phenotype induced by intracellular *Salmonella* is the massive remodeling of the endosomal system of the host cell. In addition to the recruitment of various vesicles to the SCV, the massive formation of tubular membrane compartments was observed [2]. Collectively, these compartments are referred to as *Salmonella*-induced tubules (SIT) [3, 4]. One subset of SIT, termed *Salmonella*-induced filaments or SIFs, is characterized by the presence of late endosomal/lysosomal markers such as lysosomal glycoproteins (lgp, such as LAMP1).

Inside the SCV, STM metabolism is altered and global changes in gene expression were observed [5]. One key factor is the SsrAB virulon [6, 7], a large group of genes located in *Salmonella* Pathogenicity Island 2 (SPI2) encoding a type III secretion system (T3SS), a subset of effector proteins and their chaperones, the SsrAB two-component system, and various genes outside of SPI2 encoding effector proteins of the SPI2-T3SS [8, 9]. Mutant strains defective in the SPI2-T3SS are highly attenuated in systemic disease in the murine model, as well as in intracellular survival and proliferation.

Induction of SIFs strictly depends on the active translocation of SPI2-T3SS effector proteins. Mutant strains defective in the SPI2-T3SS lack SIF formation [4]. Ultrastructural analyses revealed that SIFs induced by WT STM are composed of two membranes with ’tubule within tubule’ architecture (double membrane SIF, dm SIF), while SIFs induced by STM Δ*sseF* have thinner diameter and are composed of one membrane with single tubule architecture (single membrane SIF, sm SIF) [10]. An effector protein with central role in SIF formation is SifA. SifA-deficient STM not only lack SIF formation [11], but also show defects in maintaining the integrity of the SCV [12], resulting in increased release of bacteria into the host cell cytosol. Mutant strains lacking effector proteins SseF and SseG maintain intact SCV and show identical phenotypes including reduced intracellular proliferation and formation of aberrant SIFs [13].

We recently demonstrated that SIFs contribute to nutrition of intracellular STM within the SCV [14]. Connection of the SCV to the extensive network of tubular membrane compartments provides access to endosomal cargo and allows rapid proliferation of STM WT within the SCV. STM without SIF formation are excluded from this form of nutritional supply. We also demonstrated that lack of SseF results in reduced access to endosomal cargo [14]. Thus, we hypothesize that the nutritional conditions of intracellular STM WT are distinct from STM unable to induce SIF formation (e.g. STM Δ*ssaV*), or STM with reduced induction of SIFs (e.g. STM Δ*sseF*).

Various antimicrobial factors act on STM in the SCV, for example reactive oxygen species (ROS) and/ or reactive nitrogen species (RNS), lysosomal hydrolases or antimicrobial peptides [reviewed in 15, 16]. The connection of the SCV to a SIF network may increase exchange of compounds delivered to the SCV with luminal content of SIFs. Such exchange could reduce the concentration of antimicrobial host factors. Thus, we hypothesize that STM strains unable to induce SIF formation or showing altered architecture of SIFs are exposed to a higher degree to stress induced by antimicrobial host defenses.

To test these hypotheses, we performed proteome analyses of intracellular STM recovered from macrophages. We performed infection with STM WT, Δ*ssaV*, or Δ*sseF* for comparison of the strains with full capability to induce SIFs, absence of SIF formation, or formation of aberrant SIFs, respectively. Proteome analyses indicate individual signatures of these strains and support distinct nutritional conditions and exposure to host cell defense mechanisms as a function of SIF formation.

## Results

### Proteomic analysis of intracellular STM with distinct SIF phenotypes

**Fig 1:**
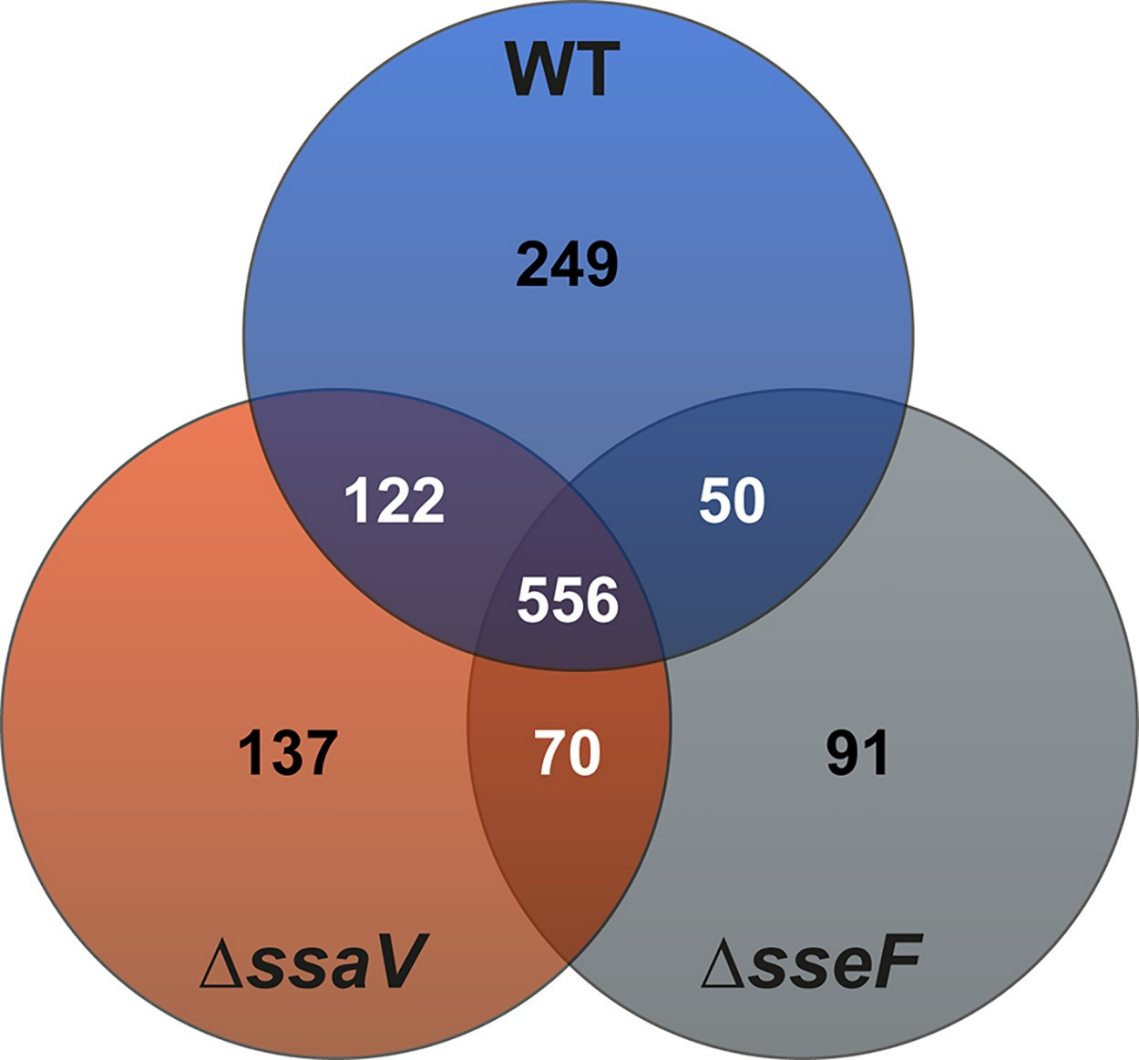
Proteomes of STM WT, Δ*ssaV* and Δ*sseF* strains isolated from RAW264.7 macrophages. Depicted are numbers of proteins detected in all strains (center), or in two strains (overlapping circles). Numbers in the center of circles indicate the uniquely identified proteins of the respective strain.

Previous studies demonstrated the contribution of SIFs to the nutritional supply of intracellular STM and additional function in dilution of antimicrobial compounds was assumed [14]. In order to test these hypotheses, we performed a proteomic analysis of STM with different SIF phenotypes, i.e. STM WT, Δ*sseF*, and Δ*ssaV*, isolated from RAW264.7 macrophages. For each biological replicate, we isolated 4 x 10^8^ to 1.9 x 10^9^ bacteria and detected 975, 884 and 766 proteins for STM WT, Δ*ssaV*, and Δ*sseF* strains, respectively. In total 1,307 distinct proteins were identified, of which 556 proteins were in common between the three strains tested (see Fig 1, S1 Table). We detected the highest overlap between WT and STM Δ*ssaV*, with 678 proteins in common, whereas proteomes of WT and STM Δ*sseF* shared 606 proteins. Uniquely identified were 249, 137 and 91 proteins for STM WT, Δ*ssaV*, and Δ*sseF* strains, respectively.

To test if the proteome profiles are the response to an altered intracellular environment rather than caused by the mutation, we analyzed the proteome of STM WT and Δ*sseF* after growth under conditions inducing the SsrAB regulon [17]. Cultures were grown in PCN minimal medium to the stage of transition into stationary phase and protein content was profiled using data-independent mass spectrometry. Altogether, 934 proteins were identified and quantified in both strains (S2 Table). No significant differences in the abundance of these proteins were detected between the *in vitro* grown STM WT and Δ*sseF*. Thus, the mutation had no discernible effects during *in vitro* growth in defined synthetic medium. With these data sets for reference, we proceeded to analyze the proteome profiles of WT and STM Δ*sseF*, isolated from RAW264.7 macrophages.

We first analyzed the transition from culture in defined synthetic medium to conditions within macrophages. A total of 242 proteins were found to be differentially abundant in STM WT from macrophages compared to growth in PCN, indicating a significant response to the intracellular environment (S3 Table). More importantly, we observed a clear induction of pathways and regulons previously associated with the intracellular lifestyle of STM. Examples include elements of the PhoPQ, OmpR, Fur, Hfq, YdcR and SlyA regulons. Thus, our data are in excellent agreement with previous studies [18–24].

Interestingly, for 115 proteins the Δ*sseF* strain showed a similar tendency of increase during macrophage infection as for WT. These proteins likely form the core of proteins involved in the adaptation to intracellular growth. Among these proteins are again well known infection-associated proteins such as SlyA/B, PhoP/N, Fur, Hfq, YdcR, SodC1 and PntA. However, almost an equal amount of proteins (127 and 123 in STM WT and Δ*sseF*, respectively) were either only differentially increased in STM WT or Δ*sseF*, or were regulated in different directions (i.e. increased in STM WT and decreased in STM Δ*sseF*, or vice versa). These large differences suggest that lack of SseF creates a somewhat altered intracellular environment, necessitating specific adaptations. This is evidenced by the reduced intracellular replication of STM Δ*sseF* and even lower of STM Δ*ssaV* in epithelial cells [25] and macrophages [13]. Since this reduced intracellular proliferation coincides with an aberrant SIF formation for STM Δ*sseF*, or in case of STM Δ*ssaV*, the loss of SIFs, the question arises whether the proteomic changes can help to pinpoint the cause of these growth retardations. Potentially, the alteration of the STM-containing compartment due to the lack of SseF and SsaV, respectively, could limit nutrient availability and/or have a higher presence of stressors compared to fully formed SIFs in the WT. To this end, we classified the surveyed proteomes of WT as well as both mutants according the Clusters of Orthologous Groups (COG) schemes and determined the relative protein abundance for each category. To account for different protein yields due to growth conditions as well as variability in injection, we normalized abundance values to the total protein abundance measured for each sample injection.

**Fig 2:**
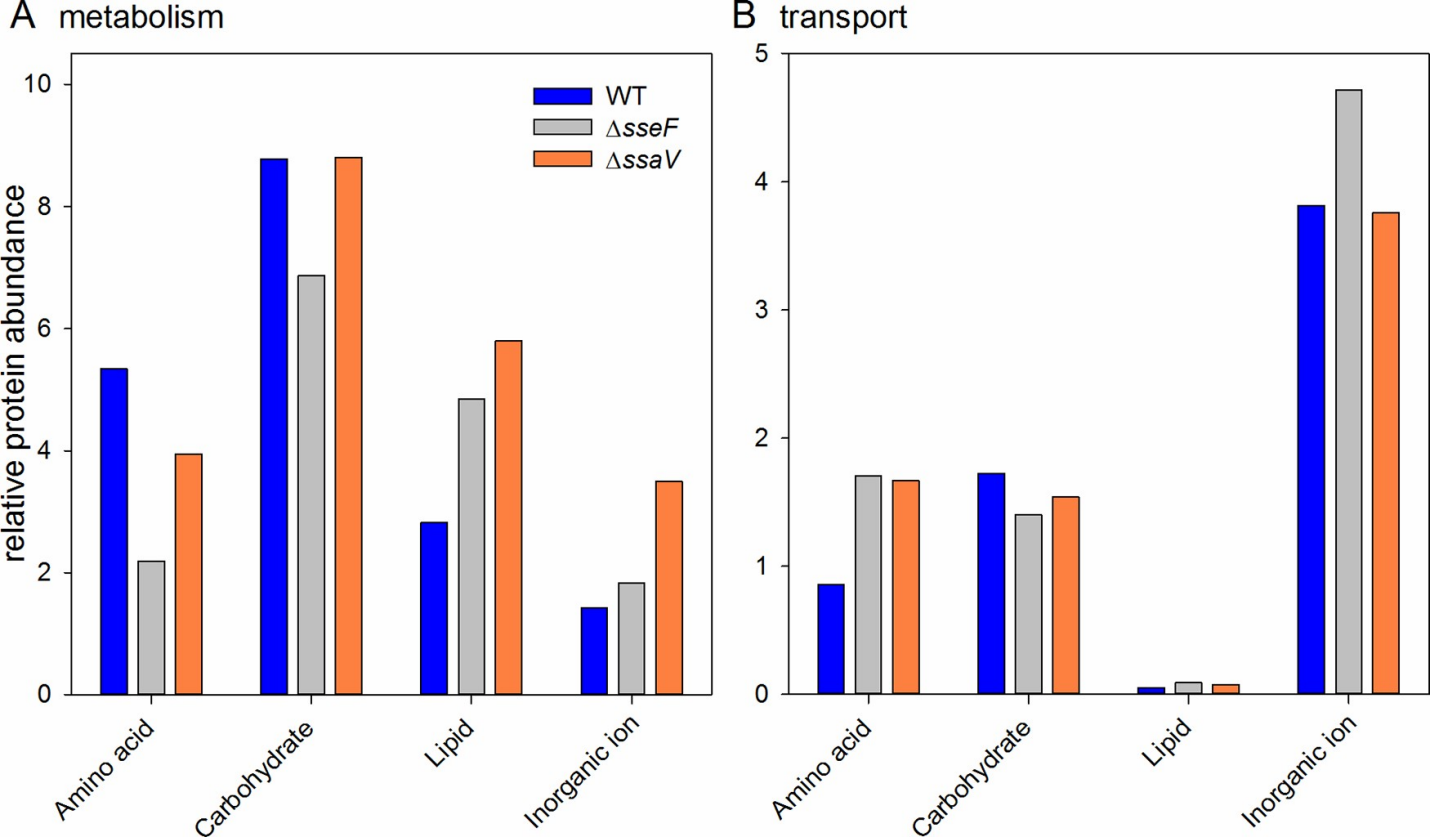
Protein classes altered in STM isolated from RAW264.7 macrophages. Depicted is for STM WT (blue), Δ*sseF* (grey) and Δ*ssaV* (orange) strains the relative abundance of all identified proteins involved in (A) metabolism or (B) transport of lipids, amino acids, carbohydrates and inorganic ions. The categories were derived from COG classifications.

We focused on metabolic functions as these provide the most insight into the availability of nutrients for the respective strains during intracellular growth and extracted proteins involved in carbon, lipid, amino acid and inorganic ion transport and metabolism. We considered transport and metabolic enzymes in each category separately in order to get a better view on which nutrients may be available for each respective strain. The COG categorization of transporters showed an increase of overall transporter abundance in STM Δ*sseF* (7.89 relative abundance, RA) and STM Δ*ssaV* (7.7 RA) compared to WT (6.4 RA). Looking closer at the different COG categories (Fig 2), it becomes apparent that the largest amount of transporters is involved in inorganic ion transport in all three strains, with STM Δ*sseF* exhibiting a larger abundance than the other strains. For amino acid and lipid transport both mutants exhibited a higher abundance of transporters than the WT, but lower abundance for carbohydrate transport.

An analysis of metabolism-related proteins revealed that overall STM Δ*ssaV* had the highest abundance of metabolism-related proteins (22 RA) compared to STM Δ*sseF* (16 RA) and WT (18 RA). The increase is mostly driven by increased proteins in the category of inorganic ion and lipid metabolism. It should be noted that proteins characterized as inorganic ion metabolism according to COG are in fact stress-related proteins, such as the superoxide dismutase SodA (increased 4.0-fold in STM Δ*ssaV*, 2.2-fold in STM Δ*sseF*) or the unspecific DNA-binding protein Dps, which is involved in protecting the DNA during starvation and oxidative stress [26].

Enzymes involved in lipid metabolism were increased in both mutants (Fig 2). For example, the PhoPQ-regulated non-specific acid phosphatase was elevated in both mutants, an indication of reduced phosphate availability in the mutants compared to the WT. Even though amino acid transport was elevated in both mutants, amino acid metabolism was mostly reduced. Relative protein abundances of proteins involved in carbon metabolism were unexpectedly similar for WT and Δ*ssaV* (8.77 and 8.8 RA, respectively) and reduced for Δ*sseF* (6.87 RA).

This overview demonstrated significant changes in the abundance of metabolic functions indicative of different growth situations in the examined strains. While in both mutants there are signatures of increased phosphate limitation compared to WT, there are also differences especially related to stress responses and carbohydrate- and amino acid metabolism, as well as membrane-lipid metabolism. Taken together, the data indicate the overall reduced activity of STM Δ*sseF*, whereas in STM Δ*ssaV* a number of metabolic shifts are connected to adaptation to stress.

### Induction of sm SIFs and dm SIFs decrease stress exposure of intracellular STM

**Fig 3:**
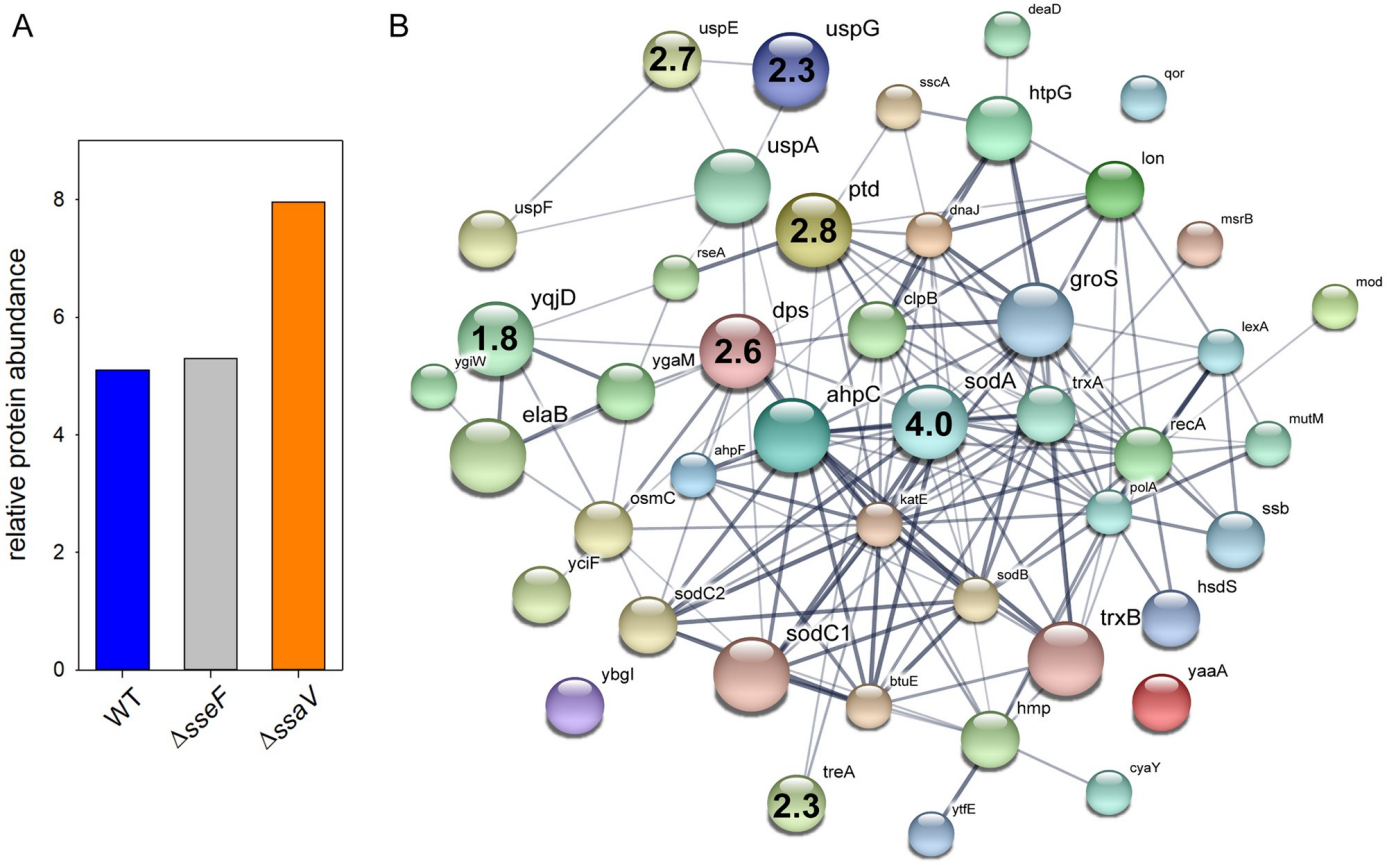
Increased abundance of stress response proteins in STM Δ*ssaV*. Detected proteins were classified according to Gene Ontology (biological process) to the group ‘response to stress’. A) The amounts of stress proteins are given as percentage of the detected proteins in total for STM WT, Δ*sseF*, and Δ*ssaV*. B) Visualization of stress response proteins detected in STM Δ*ssaV* using STRING [70]. Size of nodes indicates the amount detected for each protein. Numbers inside nodes show the ratio Δ*ssaV*/WT of proteins with significantly enhanced protein levels in the mutant strain. Statistical analysis was performed using Student’s *t*-test, *p* < 0.01 was considered as significantly different.

We hypothesized that SIF biogenesis decreases host defense-mediated stress on STM in the SCV and anticipated stronger stress response for STM Δ*ssaV* and an intermediate level for STM Δ*sseF*. Proteins detected for all strains were classified according to Gene ontology (biological process) and filtered for the group ‘response to stress’. Considering the relative protein amount of stress-related proteins we obtained similar values for STM WT and Δ*sseF* (5.1 and 5.3 RA), but elevated levels for STM Δ*ssaV* (7.96 RA), indicating an increased stress response for STM Δ*ssaV* (Fig 3A).

To determine if infection of macrophages by STM WT, Δ*sseF* and Δ*ssaV* results in similar or distinct cellular response, we analyzed the generation of ROS. Dihydrorhodamine 123 (DHR 123) is a cell-permeable dye that is oxidized by hydrogen peroxide or peroxynitrite to rhodamine 123 [27]. The resulting increase in green fluorescence can be quantified by flow cytometry on a single cell level. Infection or RAW264.7 cells by STM WT lead to increased rhodamine 123 fluorescence with about twofold increase of x-median relative fluorescence units (S1A and S1B Fig). We observed that the relative amount of ROS in infected cells was independent of the strain used. Infection of cells and addition of diphenylene-iodonium chloride (DPI), an inhibitor of ROS generation by NADPH oxidase and other enzymes [28], resulted in signal intensities as low as in non-infected cells. Infection with STM WT, Δ*sseF*, or Δ*ssaV* resulted in the same increase of rhodamine 123 fluorescence intensities (S1C and S1D Fig). We conclude that STM infection of macrophages stimulates ROS production regardless of the function of the SPI2-T3SS and effector SseF.

Next, we focused on the individual proteins and their abundance (see Table 1). Significantly more abundant in STM Δ*sseF* were DegP (= Ptd or HtrA, periplasmic serine endoprotease; 2.91-fold), Dps (DNA protection during starvation protein; 2.58-fold), HtpG (chaperone protein; 1.63-fold) and Ssb (single-stranded DNA binding protein 1; 1.59-fold), indicating an enhanced response towards oxidative stress and misfolded proteins. Proteins Lon, TrxB, ClpB, AhpC, RecA are involved in similar mechanisms and were less abundant in STM Δ*sseF* compared to WT (1.77- to 4.97-fold lower).

**Table 1:** Ratios of identified stress-response proteins in STM Δ*sseF* and Δ*ssaV* compared to WT*.

	Gene product	Protein description	Ratio mutant/WT
**altered amounts in STM** Δ***sseF***	DegP	periplasmic serine endoprotease	2.91
	Dps	DNA protection during starvation protein	2.58
	HtpG	chaperone protein	1.63
	Ssb	single-stranded DNA-binding protein	1.59
	RecA	recombinase A	0.56
	AhpC	alkyl hydroperoxide reductase C	0.52
	ClpB	chaperone protein	0.43
	TrxB	thioredoxin reductase B	0.40
	Lon	Lon protease	0.20
**altered amounts in STM** Δ***ssaV***	SodA	superoxide dismutase A	4.01
	DegP	periplasmic serine endoprotease	2.79
	UspE	universal stress protein E	2.66
	Dps	DNA protection during starvation protein	2.55
	TreA	periplasmic trehalase A	2.32
	UspG	universal stress protein G	2.28
	YqjD	inner membrane protein	1.82
	ClpB	chaperone protein	0.59
	RecA	recombinase A	0.57
	DnaJ	chaperone protein	0.47
	Lon	Lon protease	0.32

* Listed are proteins classified in category ‘stress response’ according to GO (biological process). Only proteins with significantly differential levels are depicted (Student’s *t*-test, *p* < 0.01).

We observed a different pattern for the comparison of STM WT and Δ*ssaV*. Seven proteins were significantly more abundant in the mutant strain, whereby SodA (superoxide dismutase A) was the strongest induced stress-related protein (4.0-fold). Beside Dps (2.55-fold) and DegP (= Ptd or HtrA; 2.79-fold), which were induced in STM Δ*sseF* as well, STM Δ*ssaV* showed a higher level of universal stress proteins E (2.66-fold) and G (2.28-fold), trehalase A (2.32-fold) and YqjD (1.81-fold), a protein localizing ribosomes to the membrane during stationary phase in *E*. *coli* [29] (see Fig 3B). Four proteins were significantly down-regulated, of which three were also more abundant in WT compared to STM Δ*sseF* (Lon, ClpB, RecA; 1.7- to 3.15-fold). Additionally, the chaperone DnaJ was detected in a higher abundance for WT compared to STM Δ*ssaV* (2.12-fold).

We conclude that dm SIFs induced by STM WT and sm SIFs induced by STM Δ*sseF* both result in reduced stress exposure of intracellular STM, while lack of SPI2-T3SS effector protein translocation and SIF biogenesis result in increased exposure to hostile intracellular environments.

### Analyses of stress reporter fusions in STM on single cell level

**Fig 4:**
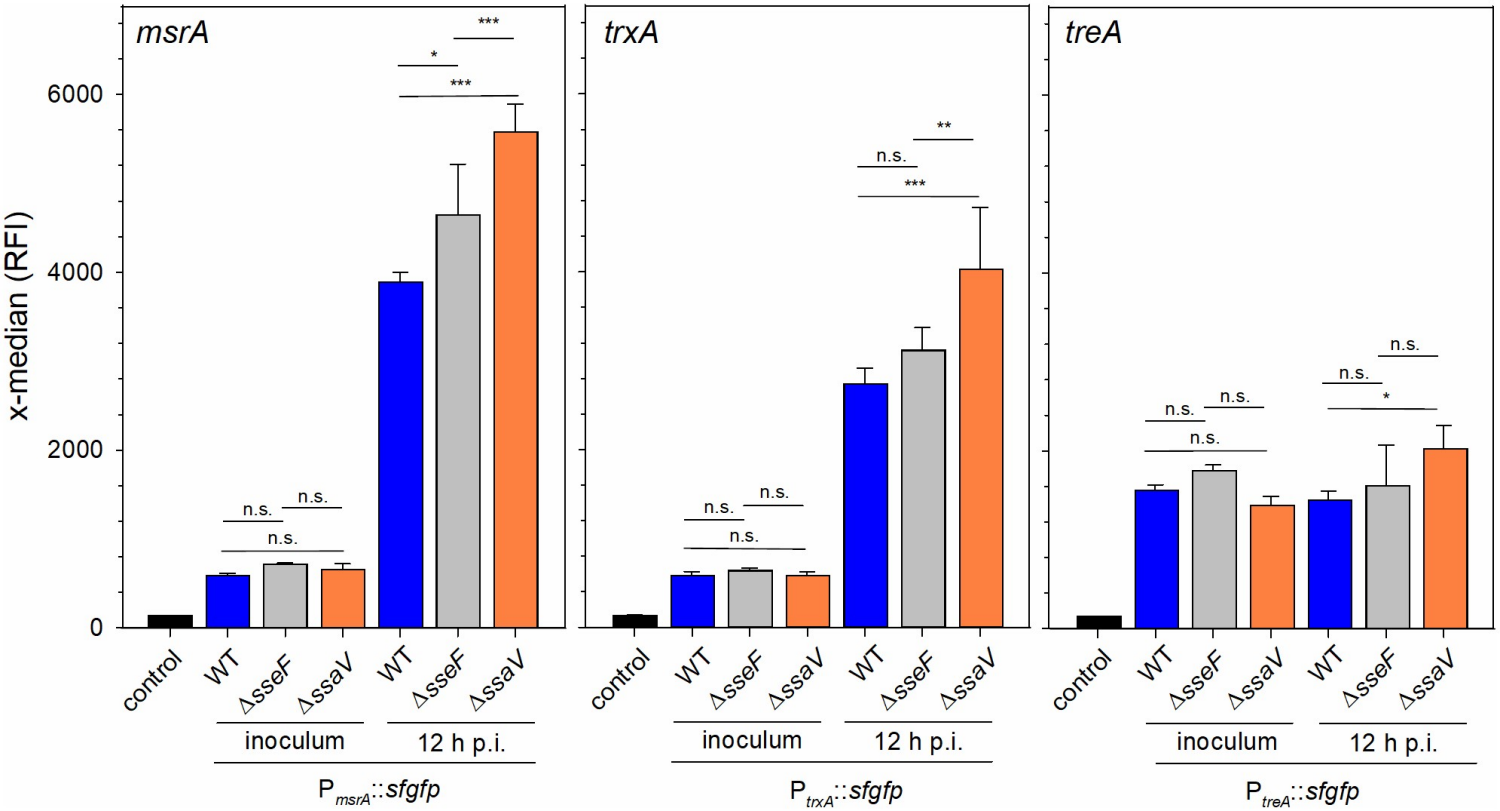
Differential expression of stress response functions. STM WT, Δ*sseF* or Δ*ssaV* strains harboring dual color fluorescence reporter for *msrA*, *trxA* or *treA* were cultured in LB o/n. Aliquots of the o/n culture were fixed and subjected to flow cytometry. RAW264.7 macrophages were infected with reporter strains, cells were lysed 12 h p.i., bacteria were recovered and subjected to flow cytometry. The gating for individual bacteria and quantification of induction is described in detail in S2 Fig. Means and standard deviations of three independent experiments are shown. Statistical analysis was performed by One-way ANOVA and significance levels are indicated as follows: *, *p* < 0.05; **, *p* < 0.01; ***, *p* < 0.001; n.s., not significant.

To further corroborate the role of the manipulation of the host cell endosomal system and the degree of stress exposure and response by intracellular STM, we deployed dual color fluorescence protein reporter analyses. The encoded proteins were identified directly, or as part of a response regulon, as increased in intracellular STM Δ*sseF* and Δ*ssaV* compared to STM WT. *trxA* encodes cytoplasmic thioredoxin, an enzyme reducing cysteine after radical-induced oxidization, *msrA* encodes a methionine reductase required for repair of damaged methionine, and *treA* encodes a trehalase that supports adaptation to osmotic stress. The promoters of these genes were used to generate sfGFP reporter fusions on plasmids that also encoded constitutively expressed DsRed. We analyzed the expression of the various reporter fusions in STM WT, Δ*sseF* and Δ*ssaV* background on the level of single intracellular bacteria recovered 12 h after infection from RAW264.7 macrophages (S2 Fig). For comparison, the expression levels were determined for bacterial strains grown o/n in LB for preparation of the inoculum. In WT, Δ*sseF*, and Δ*ssaV*, expression of *msrA* and *trxA* reporters was highly induced in intracellular bacteria compared to the inoculum (Fig 4). Expression levels of the *treA* reporter did not increase in intracellular bacteria, indicating the similar stress levels are present under extracellular and intracellular conditions. With focus on expression by intracellular STM, we observed that signals of all three reporters was highest in STM Δ*ssaV*, while expression in Δ*sseF* background was increased compared to WT, but lower than in STM Δ*ssaV*. These results obtained for single intracellular bacteria indicate the increased expression of stress response mechanisms in mutant strains with aberrant or absent ability in manipulation of the host endosomal system. This induction may reflect the increased exposure to host cell defense mechanisms such as ROS generation.

We assessed the effect of ROS generation on intracellular proliferation of STM WT, Δ*ssaV* and Δ*sseF* in RAW264.7 macrophages (S3 Fig). After infection, cells were treated with phenylarsine oxide (PAO) or DPI, or left untreated. While intracellular proliferation of STM Δ*sseF* and Δ*ssaV* was reduced compared to STM WT in non-treated cells, the inhibition of ROS generation fully abrogated the defects of the mutant strains (S3 Fig). Our data indicate that STM infection of macrophages induces ROS generation (S1 Fig), and ROS affect intracellular survival and replication of STM Δ*sseF* and Δ*ssaV* more severely than STM WT.

### Global down-regulation of carbon and amino acid metabolism in Δ*sseF* and Δ*ssaV* strains

**Fig 5:**
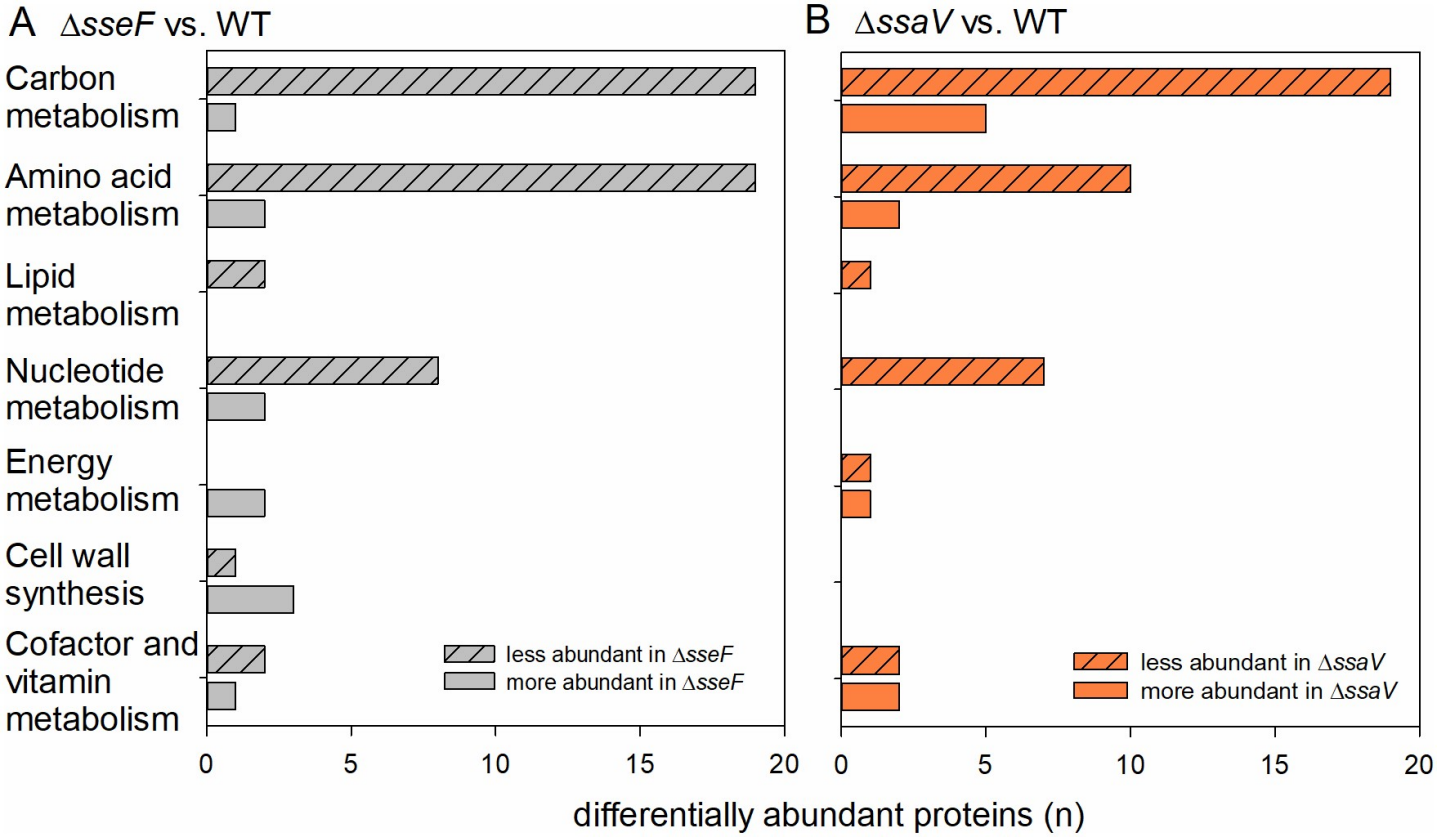
Differential abundance of metabolic proteins in intracellular STM Δ*sseF* and Δ*ssaV* compared to WT. Depicted are numbers of proteins with significantly increased (solid bars) and decreased (hatched bars) abundance in metabolic pathways for STM Δ*sseF* (A) and STM Δ*ssaV* (B) compared to WT according to KEGG. Statistical analyses were performed using Student’s *t*-test, *p* < 0.01 was considered as significantly different.

Recent work demonstrated that SIFs have a critical role in the intracellular nutrition of STM [14]. As the analysis of RA regarding proteins involved in metabolism led to the unexpected results, we focused on differentially regulated proteins involved in the central carbon and amino acid metabolism of STM WT, Δ*sseF* and Δ*ssaV* and classified the identified proteins according to the Kyoto Encyclopedia of Genes and Genome (KEGG) [30] (Fig 5). 19 proteins of the carbon and amino acid (AA) metabolism had lower levels in Δ*sseF* compared to WT STM. Similar tendencies were obtained for STM Δ*ssaV*.

**Fig 6:**
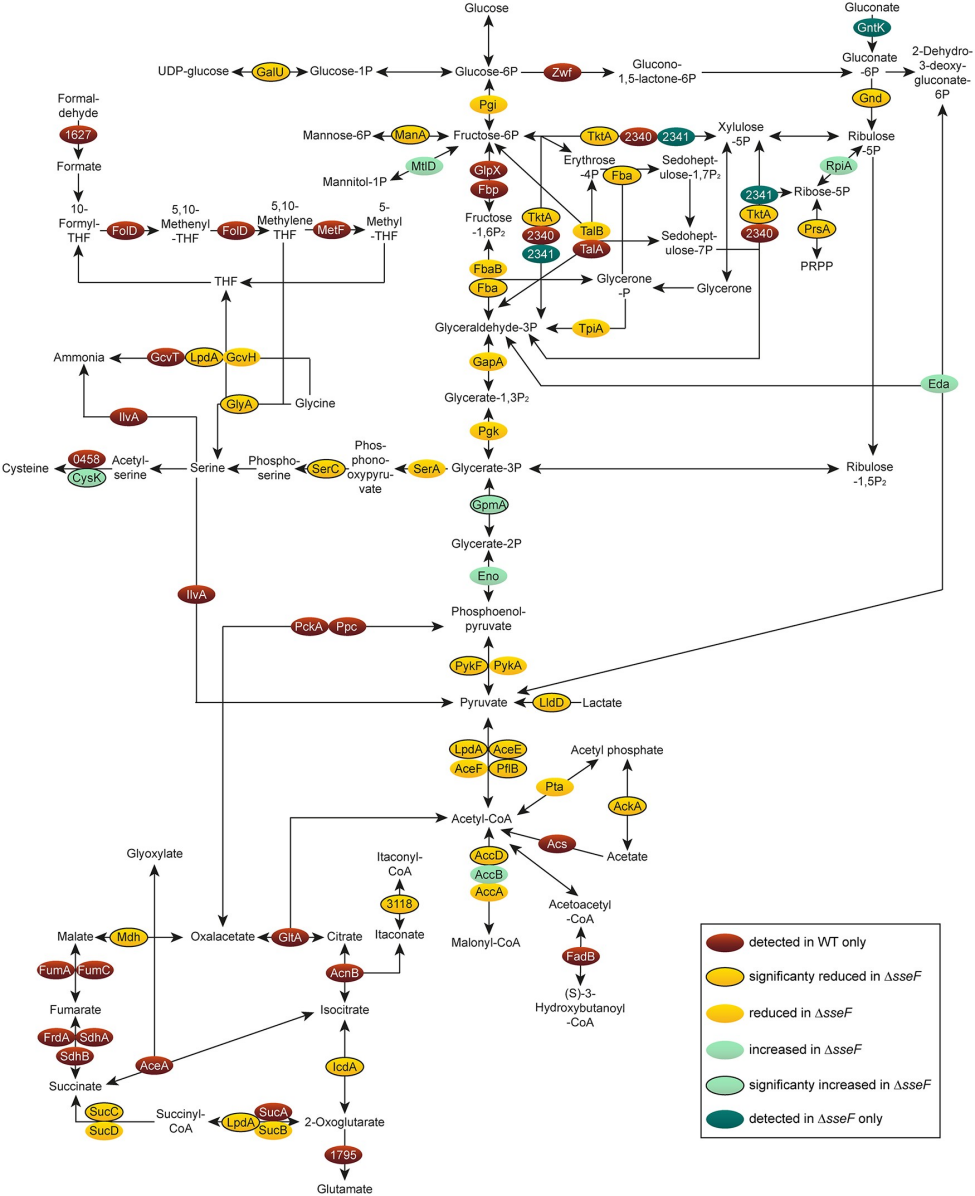
Central carbon metabolism of intracellular STM Δ*sseF* compared to WT. Detected proteins were mapped against pathways annotated as involved in the central carbon metabolism of STM according to KEGG. The enzymes are presented in ovals; distinct colors indicate the abundance of the specific protein in the Δ*sseF* strain compared to STM WT. For statistical analyses, only proteins detected in at least two replicates were considered. Statistical analysis was performed as indicated in Fig 3.

**Fig 7:**
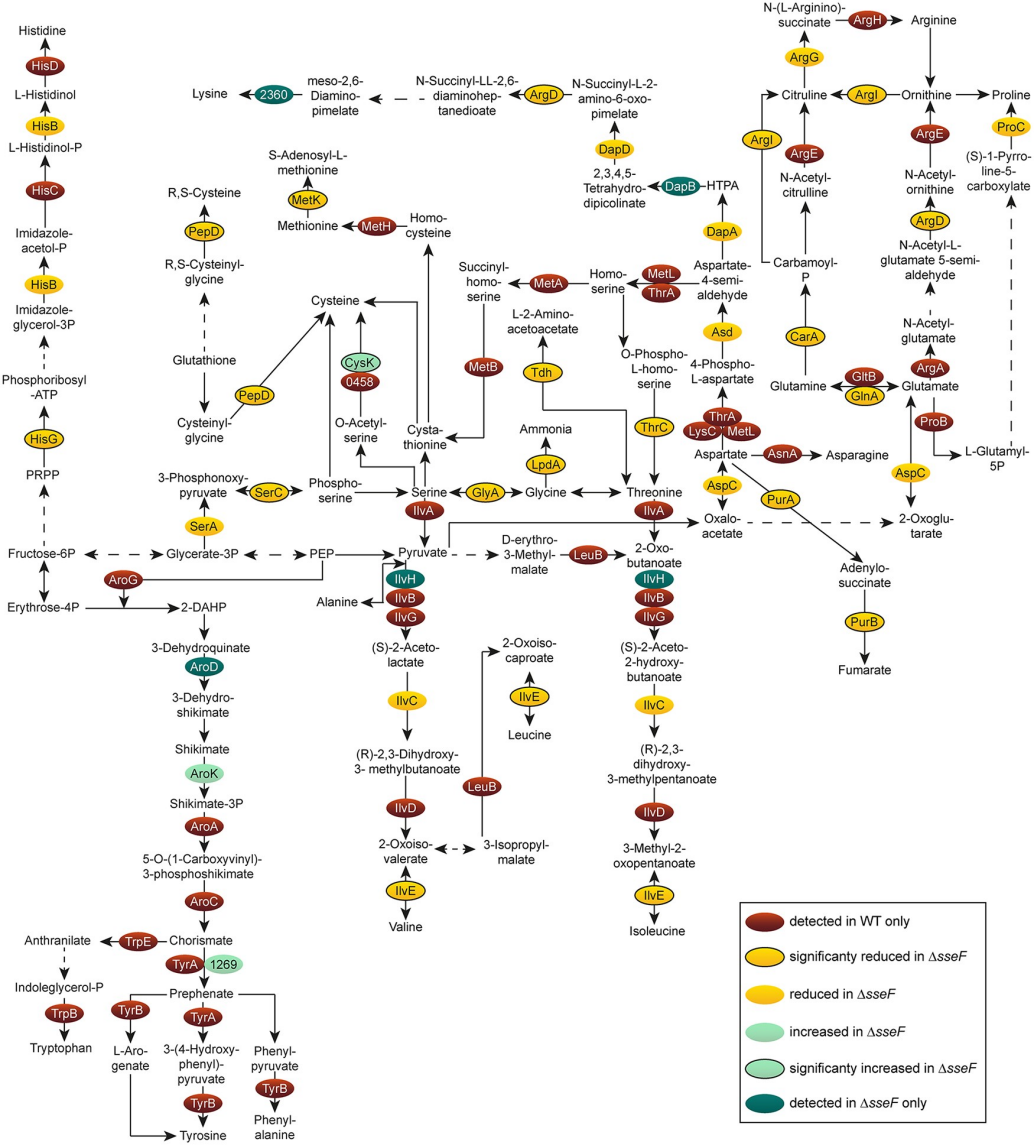
Amino acid metabolism of intracellular STM Δ*sseF* compared to WT. Detected proteins were mapped against the pathways annotated as involved in the central amino acid metabolism of STM according to KEGG. The enzymes are presented in ovals; distinct colors indicate the abundance of the specific protein in STM Δ*sseF* compared to STM WT. Statistical analysis was performed as indicated for Fig 3.

We analyzed if central carbon metabolism (CCM) including glycolysis, pentose phosphate pathway (PPP) and TCA cycle was modulated in STM Δ*sseF* and Δ*ssaV*. Both mutant strains showed a high number of proteins with reduced abundance of all CCM pathways compared to STM WT (Fig 6, S4 Fig), as well as reduced AA metabolism (Fig 7, S5 Fig). Many enzymes were only identified for STM WT, indicating very low levels in STM Δ*sseF*. Especially proteins involved in synthesis of tryptophan, tyrosine and phenylalanine were only found in STM WT.

To validate the down-regulation of AA metabolism, we performed qPCR analyses. RNA of pooled bacterial pellets from large-scale infection experiments with STM WT and the Δ*sseF* strain was isolated. RNA isolated 12 h p.i. did not reveal differential expression, using RNA from STM isolated 10 h p.i. accompanied our proteomic data. Results of qPCR of *glyA* (serine and glycine metabolism) confirmed proteomics data (S6 Fig).

Whereas a high number of proteins involved in CCM and AA metabolism were reduced in their abundance in STM Δ*sseF* compared to WT, specific proteins were up-regulated in a significant manner: GpmA (phosphoglyceromutase) was slightly increased (1.38-fold), and CysK (cysteine synthase A) was 1.8-fold more abundant. Interestingly, these proteins were up-regulated even higher in STM Δ*ssaV* compared to WT, i.e. 1.7- and 2.56-fold for GpmA and CysK, respectively. Thus, we anticipate regulation mechanisms and roles of these two enzymes during nutrient limitation to be distinct from all other metabolic proteins detected in our approach.

### Increased levels of ABC transporter and PTS subunits in *sseF*- or *ssaV*-deficient STM

**Fig 8:**
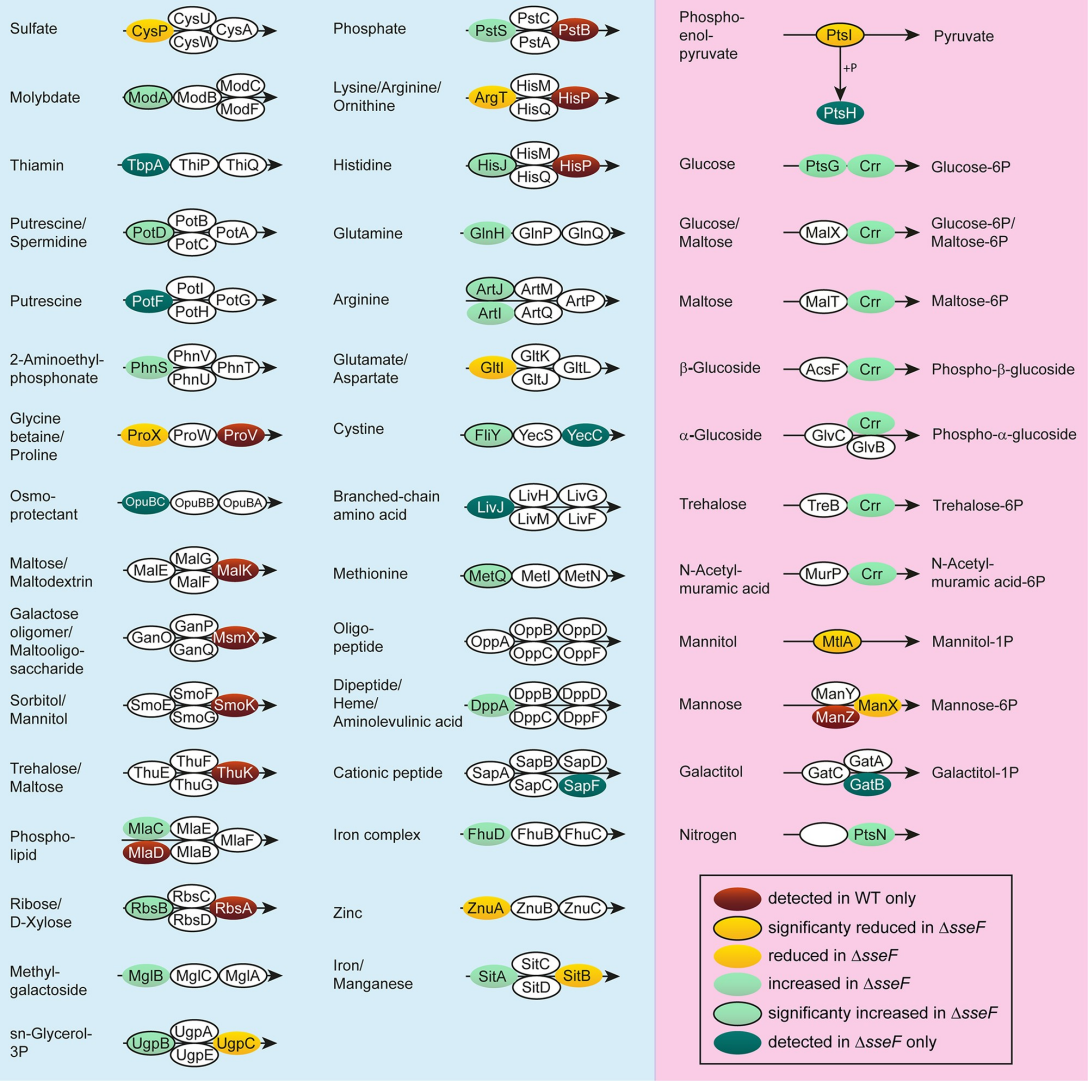
Levels of ABC transporter and phosphotransferase system (PTS) proteins in *sseF*-deficient STM. Analyses of components of ABC transporters (blue background) or PTSs (pink background) detected in intracellular STM WT and Δ*sseF* according to KEGG. Subunits are presented in ovals; distinct colors indicate the abundance of the specific protein in the Δ*sseF* strain compared to STM WT. Statistical analysis was performed as indicated for Fig 3.

Successful adaptation of STM to intracellular life demands expression of transporters for access to host-derived nutrients [5]. As analyses of RA demonstrated increased and decreased amounts of proteins involved in amino acid and carbohydrate transport, respectively, in the mutant strains, we interrogated our proteomic data for presence of subunits of ABC transporters and phosphotransferase systems (PTS) with significantly changed amounts in STM WT, Δ*sseF* and Δ*ssaV* (Fig 8, S7 Fig). We detected significantly enhanced levels of eight ABC transporter subunits in Δ*sseF* compared to STM WT (1.41- to 3.19-fold). These transporters are responsible for the uptake of amino acids (arginine, cysteine, histidine and methionine), ions (molybdate, putrescine, spermidine), and monosaccharides (sn-glycerol-3-P). If including proteins only detected in STM Δ*sseF*, or with non-significantly increased levels compared to STM WT, 24 ABC transporter subunits were identified. ABC transporter subunits with significantly less abundant levels were not detected for STM Δ*sseF*. 16 proteins for ABC transporter subunits were only detected in STM WT or had non-significantly lower levels in STM Δ*sseF*. We confirmed the induction of two subunits of ABC transporters for methionine and arginine, i.e. *metQ* and *artJ*, by qPCR (S6 Fig).

For PTS, we observed in STM Δ*sseF* significantly reduced abundance of PtsI and MtlA that are important for uptake of phosphoenolpyruvate (PEP) and mannitol, respectively. Furthermore, transporters involved in uptake of glucose (PtsH) or galactitol (GatB) were only detected in the mutant strain. PtsG and PtsN were increased in the proteomic data set in a non-significant manner. Crr, important for the uptake of several members of the glucose family (glucose, maltose, trehalose, glucosides and N-acetyl-muramic acid) was increased by a factor of 1.38.

Regarding levels of ABC transporter and PTS systems, STM Δ*ssaV* showed a pattern similar to STM Δ*sseF* (see S7 Fig). The abundance of 23 ABC transporter subunits was increased in STM Δ*ssaV* compared to WT, even though only significantly different for three proteins. Furthermore, we determined higher levels of 6 PTS subunits, including significantly increased amounts of Crr (2.01-fold) (S7 Fig).

Taken together STM Δ*sseF* and Δ*ssaV* showed increased levels of proteins involved in the transport and incorporation of nutrients important for intracellular growth.

## Discussion

This work provides the first systematic analysis of the impact of SPI2-T3SS-mediated endosomal remodeling of host cells on the global physiology of intracellular STM. For this, we performed quantitative proteomics of intracellular STM isolated from RAW264.7 macrophages and compared STM WT to highly attenuated STM Δ*ssaV* defective in the SPI2-T3SS, and moderately attenuated Δ*sseF*, defective in a single SPI2-T3SS effector required to establish the full extent of remodeling of the host cell endosomal system. The analysis revealed distinct proteomic signatures for nutritional stress and compensatory regulation in both Δ*sseF* and Δ*ssaV* strains that is discussed in detail below. In addition, the Δ*ssaV* strain specifically revealed proteomic signatures of increased exposure to stress imposed by host cell antimicrobial defense mechanisms.

### Starvation stress response in STM Δ*sseF* and STM Δ*ssaV*

STM is a ‘metabolic all-rounder’ [31, 32] with the ability to use a wide range of C-sources during life inside host cells [5, 32]. Previous results obtained from proteomic analysis of intracellular STM isolated from RAW264.7 macrophages, epithelial cell lines and mouse spleen indicated that a broad range of metabolic pathways is active [5, 33–35]. Our analyses also detected in STM WT proteins of all central pathways (S8 Fig) and indicated that our infection models and proteomic analyses are comparable to prior results. The reduced abundance of proteins involved in metabolic processes in STM Δ*sseF* and Δ*ssaV* indicates a global down-regulation of AA metabolism and CCM. Former work suggested that remodeling of the host cell endosomal system requires function of SsaV and SseF, and that this remodeling is important for the intracellular nutrition of STM [14, 36]. Thus, the global down-regulation of metabolic pathways indicates nutritional limitations for STM Δ*sseF* and Δ*ssaV*.

The first response to limited amounts of C-sources such as glucose and AA are increasing concentrations of cAMP and the alarmones pppGpp and ppGpp (further referred to as (p)ppGpp). The cAMP-CRP complex activates the expression of many enzymes involved in the CCM and represses expression of CRP [37]. The reduced abundance of CRP in STM Δ*sseF* (2.22-fold, see S1 Table) thus is an indicator for cAMP-CRP regulation at the onset of starvation. The reduction of CRP in STM Δ*ssaV* was less pronounced and this may indicate the different time course of nutritional limitation, and for the Δ*ssaV* strain onset of starvation is expected much earlier than 12 h p.i, the time point for sampling for proteomics.

In the course of stringent response, RelA and SpoT synthesize (p)ppGpp. High (p)ppGpp levels lead to up-regulation of AA, carbon, and lipid metabolism, and down-regulation of translation machinery [38]. However, in *E*. *coli* suffering C-starvation, the cAMP level is rising only in the first hour, and later it decreases [39]. The same observation has been made for (p)ppGpp levels. If enhanced biosynthesis and proteolytic degradation do not restore AA levels, (p)ppGpp levels decrease [40, 41]. The nutrient deprivation of STM Δ*ssaV* and Δ*sseF* would lead to down-regulation of metabolic proteins, with early or late onset in Δ*ssaV* and Δ*sseF*, respectively.

A third metabolic adaptation observed for STM Δ*ssaV* and STM Δ*sseF* is the increased abundance of high-affinity ABC transporters that are expressed if specific AA are limiting [42, 43]. These transporters are not known to be regulated by (p)ppGpp, but by distinct regulators such as ArgR and MetR.

The metabolic adaptations of STM Δ*ssaV* and Δ*sseF*, i.e. down-regulation of AA metabolism and CCM, and up-regulation of high-affinity transporters, are characteristics of response to limiting nutrient availability. The reduction of metabolic activity and translation occurs in order to save remaining energy and peptide resources for adaptation to long periods of starvation, while upregulation of ABC transporters allows access to external nutrient pools, if environmental conditions become more favorable [44, 45]. Thus, we expect STM Δ*ssaV* and Δ*sseF* to undergo starvation and induction of the starvation stress response (SSR) during presence in the SCV in macrophages. In contrast, the induction of SIFs with large interconnected volume and double membrane architecture of tubules by STM WT leads to sufficient nutritional supply of bacteria in the SCV.

### SIF biogenesis is a unique strategy for nutrition of an intracellular pathogen

The intracellular nutrition strategy of STM is the induction of vesicle fusions to the SCV and the formation of the extensive interconnected network of SIFs [14]. Other pathogens such as *C*. *trachomatis* also highjack host cells endosomes to obtain nutrients, but the resulting PCV exhibits distinct features, such as inclusion, a single PCV with large number of *C*. *trachomatis* cells. Complementary to the role of SIF formation for the growth of intracellular STM previously reported on a single cell level [14], this work shows, by a population-based approach, the bacterial response to different degrees of nutritional limitation. Both STM Δ*ssaV*, unable to induce SIF formation, and STM Δ*sseF*, inducing SIF network with reduced volumes show proteomic signatures of nutrient starvation and the compensatory increase of uptake systems for limiting nutrients. The combination of SIF biogenesis and the metabolic flexibility of STM allow proliferation in host cells, such as macrophages, for long periods of time. If host cell nutrients are exploited and a maximal number of intracellular bacteria are reached, escape from host cells with the lowest level of damage by host defense mechanisms occurs, although the molecular mechanisms of such ‘exit strategies’ are not known in detail. Another part of the virulence strategy of STM is the formation of persisters upon nutritional limitation and exposure to antimicrobial defense mechanisms [46]. This ability allows STM intracellular survival, possibly spread within hosts, and reentering rounds of proliferation once growth restriction is released.

### Response to host defense requires a SIF network but is independent from its architecture

Although the metabolic status of STM Δ*sseF* and Δ*ssaV* appears to be similar under the conditions tested, intracellular proliferation of STM Δ*ssaV* is highly reduced compared to STM Δ*sseF*. Action of the SPI2-T3SS reduces exposure of STM to antimicrobial host functions by redirection of NADPH oxidase [47–49], iNOS [50], and reduced delivery of active cathepsins [51]. In addition, the formation of a tubular SIF network was suggested to reduce exposure of STM in the SCV to antimicrobial effector mechanisms of the host cell [14]. This function could result from dilution of antimicrobial effectors in the SCV if connections to the SIF network are established. Our proteomic analyses indicate strongly enhanced abundance of proteins related to stress response in STM Δ*ssaV*, while STM Δ*sseF* and WT exhibited lower levels with similar patterns. The ability to cope with attacks of antimicrobial defense systems of the host is indispensable for successful intracellular survival and replication, especially for pathogens residing in macrophages, being exposed to high levels of reactive oxygen and nitrogen species (ROS, RNS). STM and other intracellular pathogens have evolved various strategies to counteract antimicrobial compounds such as ROS. Upregulation of universal stress proteins, catalases, SODs and alkyl hydroperoxide reductase under oxidative stress was demonstrated for *L*. *monocytogenes* [52], mycobacteria [53], *F*. *tularensis* [54], *L*. *pneumophila* [55], STM [56] and others.

The inability to neutralize various harmful compounds often leads to attenuation. Deletion of genes of the *kai* operon involved in stress response of *L*. *pneumophila*, led to reduced growth in *Acanthamoeba castellanii* [57]. *hfq* deletion represses stress response regulators in *Shigella* spp. and led to attenuation [58]. Deletion of OxyR, a key regulator for the response to oxidative stress also in STM, and catalase G leads to attenuation of *F*. *tularensis* in the murine infection model [59]. STM defective in the ABC efflux pump MacAB has a strongly decreased replication rate in J774.16 macrophages [60] and a mutant strain defective in methionine sulfoxide reductase shows reduced proliferation in interferon γ-activated RAW264.7 macrophages [61]. Thus, the response to harmful conditions is an important virulence function of bacteria like STM.

Several studies focused on the importance of SPI2-T3SS activity for survival of the oxidative burst in immune cells. The SPI2-T3SS-dependent evasion of NADPH oxidase was reported [47], and explained by interference with assembly of functional NADPH-oxidase on the SCV by function of the SPI2-T3SS [48, 49]. Aussel et al. [56] used a ROS-dependent reporter in STM and did not observe an effect of SPI2-T3SS on ROS exposure by STM in macrophages. In contrast, van der Heijden et al. [62] demonstrated increased redox stress of a mutant strain deficient in SPI2-T3SS subunit SsaR in THP-1 cells. ROS damage DNA in intracellular STM and defects in base-excision repair system *nth*/*nei* resulted in decreased intracellular survival comparable to the attenuation of the SPI2-T3SS-deficient STM [63]. Recognition of outer membrane proteins by sensor SCAM was shown critical for activation of NADPH oxidase in macrophages, the STM WT and a SPI2-T3SS translocon-deficient strain stimulated similar activation [64].

Our data are in line with a model that similar stimulation of ROS is induced by STM WT and mutant stains in *ssaV* or *sseF*. If SPI2-T3SS-dependent redirection of vesicular transport and remodeling of the endosomal system is initiated, the exposure of STM to ROS is reduced and ROS-induced damages are ablated. Thus, interpretation of effects of ROS should consider the dynamics of the host-pathogen interplay. While stimulation and initial effects of defense mechanisms may not be controlled by STM, the later exposure to ROS is affected by the manipulation of the host cell.

Liss et al. calculated that induction of sm SIFs led to a luminal volume of the SIF network of about 50% of that of dm SIFs [14]. The data reported here indicate that proteomes of STM Δ*sseF* have the same abundance of stress related proteins as WT [14]. However, the luminal content of WT in SCV without connection to SIFs was calculated to be 34-fold decreased compared to STM Δ*sseF* inducing sm SIFs [14], thus sm SIFs could already lead to sufficient dilution of antimicrobial activities.

We hypothesize that the increased stress level STM Δ*ssaV* is exposed to results from both, the disability to avoid recruitment of iNOS and NADPH oxidase to the SCV membrane, and the strongly reduced volume of the continuum STM Δ*ssaV* resides in, leading to reduced luminal interchange and dilution.

At the current state of art, proteomics of intracellular pathogens is not possible on single cell level. Rather, amounts of 10^9^ recovered STM cells were required to reach sufficient coverage and reproducibility in quantitative proteomics. It is important to consider that population-based analyses are generally affected by the heterogeneity of phenotypes of intracellular STM. For example, STM WT may form actively replicating subpopulations, while other individual cells enter a persister state or fail to activate the proper set of virulence factors and are killed by the host cell [46, 65]. We anticipate that proteomic profiles contain mixtures of different subpopulations resulting in leveling of protein amounts. In turn, the differences in amounts of proteins involved in nutrition or stress response may actually be more pronounced in specific subpopulations. Thus, future analyses should interrogate the levels of candidate proteins in correlation with the physiological state on a single cell level. Single cell analyses will also be important to understand the orchestration of metabolic adaptation and stress management of intracellular STM by means of SIF formation. Such analyses could involve the time-resolved analyses of reporter activities for individual genes, or global analyses such as RNA-seq on single cell level [65].

**Fig 9:**
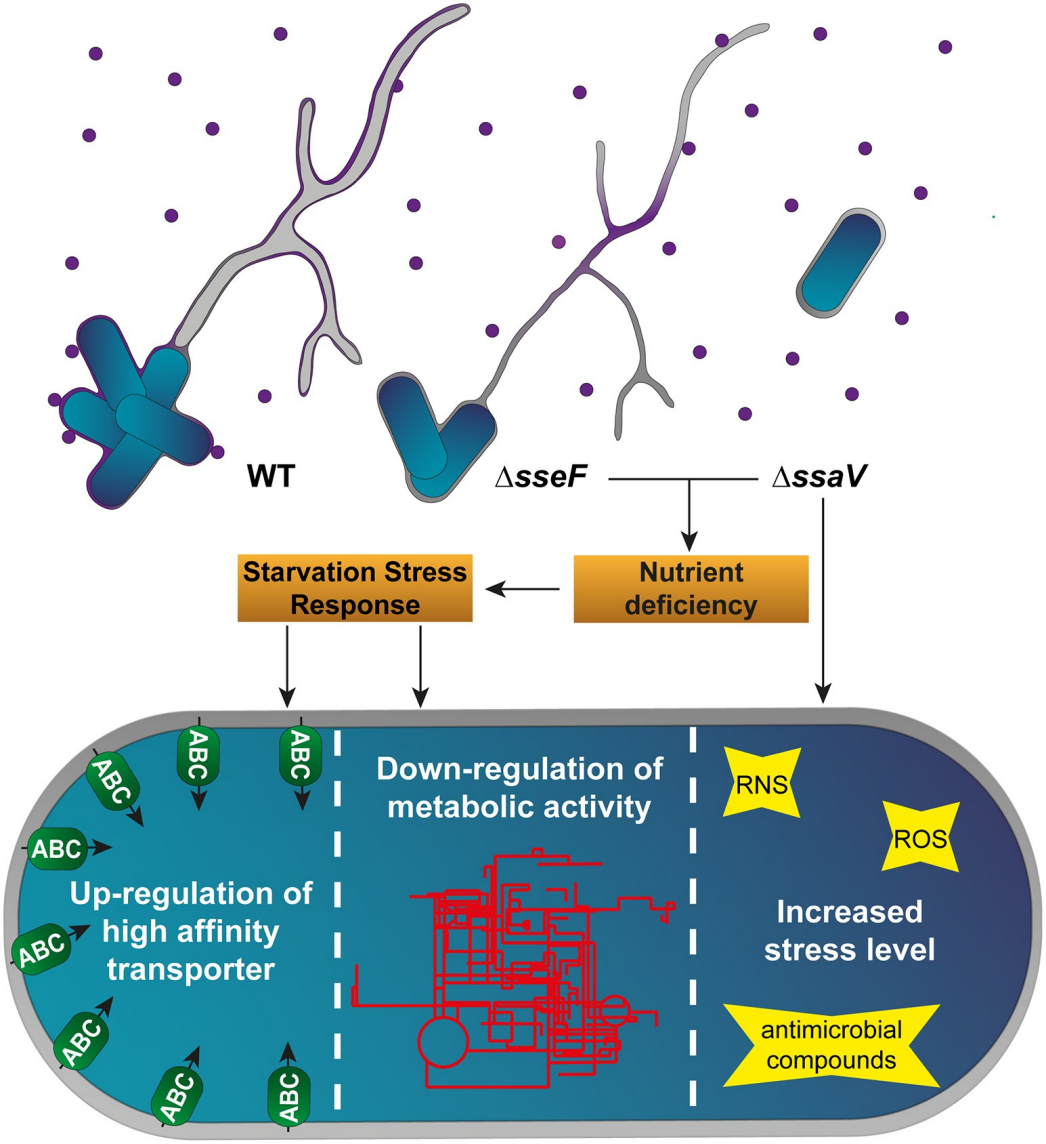
Model of the physiological adaptations of STM WT, Δ*sseF* and Δ*ssaV*. STM WT induces SIFs composed of double membranes, whereas deletion of the SPI2 effector protein SseF to T3SS subunit SsaV leads to induction of single membrane SIFs or absence of SIFs, respectively. Nutrient-containing vesicles (violet circles), fusing with the SCV and SIF network, supply intracellular STM with sufficient amounts of nutrients inside the SCV if connected to dm SIFs, but not in the case of sm SIFs. The resulting nutrient deficiency leads to induction of the starvation stress response in STM Δ*sseF* and Δ*ssaV*, which includes the up-regulation of high-affinity transporters (green ellipses) and a global down-regulation of metabolic pathways (red pattern). Thus, dm SIF architecture is obligatory for a sufficient nutritional supply of STM, but not for effective reduction of stress levels STM is exposed to within macrophages.

In conclusion, based on our proteomics datasets, we propose that i) induction of SIFs, but also the formation of dm SIFs is required for sufficient nutritional supply, ii) that sm SIFs and dm SIFs are sufficient to cope with the antimicrobial activities of the host cell, and iii) that nutritional limitations reduce the replication of STM Δ*sseF* slightly, whereas the additional inability to neutralize host defense mechanisms leads to the strongly attenuated phenotype of STM Δ*ssaV* (summarized in Fig 9). Efficient manipulation of the host cell endosomal system by SPI2-T3SS effector proteins contributes to nutrition as well as to resistance against antimicrobial host defense mechanisms. Proteomics analyses presented here support and extend the previously proposed role of SIF formation for the intracellular lifestyle of STM in mammalian host cells.

## Materials and methods

### Bacterial strains

*Salmonella enterica* serovar Typhimurium NCTC12023 was used as wild-type strain (WT). Isogenic mutant strains MvP1890 (Δ*ssaV*::FRT, defective in SPI2-T3SS apparatus) and MvP1980 (Δ*sseF*::FRT, defective in SPI2-T3SS effector protein SseF) were constructed by λ Red-mediated mutagenesis [66]. Primers required for mutagenesis, removal of resistance cassettes and control of proper insertion are listed in S4 Table. Transfer of mutant alleles into fresh strain background was mediated by P22 transduction, and *aph* resistance cassettes were removed by FLP-mediated recombination as described [36]. Bacterial strains were routinely cultured in LB Luria broth at 37°C overnight (o/n) using a roller drum at 60 rpm.

### Culture conditions for proteome profiling of STM strains in SPI2-inducing minimal medium

STM WT and Δ*sseF* were grown o/n in minimal medium (PCN minimal medium, pH 7.4, 1 mM PO_4_^-^) [36]. Cells of 1 ml culture were pelleted by centrifugation and washed thrice with SPI2-inducing medium (PCN minimal medium, pH 5.8, 0.4 mM PO_4_^-^). The pellet was resuspended in SPI2-inducing medium and the optical density was determined. 25 ml SPI2-inducing medium was inoculated to an OD_600_ of 0.01. Bacteria were cultured in a shaking water bath at 37°C for 5 h. OD_600_ was determined and 6 x 10^9^ bacteria were pelleted by centrifugation. Supernatant was removed, and the bacterial pellets frozen in liquid nitrogen.

### Infection of RAW264.7 macrophages and isolation of intracellular bacteria

To gain sufficient amounts of bacteria from infected host cells, infection parameters described by Popp et al. [36] were adjusted. Increased MOI, infection times, and centrifugal fields were compared in gentamicin protection assays, before being applied to proteome profiling of intracellular STM (see S9 Fig). Modifications of the protocol did not affect SPI2-dependent replication.

Finally, for proteomics analyses 8 x 10^6^ RAW264.7 macrophages (obtained from CLS Heidelberg, Germany) per flask were seeded into 16 cell culture flasks (75 cm^2^, TPP) one day before infection, or at 4 x 10^6^ cells per flask two days before infection. RAW264.7 macrophages were infected with a MOI of 25 with STM strains. Infection assays were centrifuged at 1,250 *x g* for 5 min., infection proceeded for 45 min. at 37°C in an atmosphere of 5% CO_2_. Infected cells were washed thrice with PBS and remaining extracellular bacteria were killed by incubation in cell culture medium containing 100 μg x ml^-1^ gentamicin for 1 h. Afterwards, the medium was exchanged to cell culture medium containing 10 μg x ml^-1^ gentamicin for further 11 h.

During isolation of intracellular bacteria, samples were stored continuously on ice and centrifugation occurred at 4°C. The infected cells were washed thrice with pre-warmed PBS. Cells were lysed by addition of 5 ml 1% Triton X-100 in PBS for 10 min. at 4°C. Cell lysates were pooled and centrifuged for 5 min. at 300 *x g*. The supernatant was centrifuged again for 10 min. at 20,000 *x g*. Pelleted bacteria were resuspended in PBS containing 0.05% SDS and 0.5% desoxycholate and centrifuged again for 10 min. at 20,000 *x g*. The washing step was repeated, and the pellet resuspended in 1 ml PBS. Bacteria were pelleted again for 10 min. at 20,000 *x g*, the supernatant discarded, and samples were stored at -80°C.

### Protein isolation of *in vitro* cultures and intracellular STM and LC-MS measurement

Proteins from bacteria were isolated using Trizol (Thermo Fisher Scientific), following the manufacturers protocol. Bacterial pellets were therefore resuspended in 750 μl Trizol and incubated at RT for 10 min. After addition of 150 μl chloroform and 3 min. incubation at RT, samples were centrifuged at 12,000 *x g* for 15 min. to isolate the organic phase. To remove DNA contamination, 225 μl of 100% ethanol were added to the organic phase. After 3 min. incubation at RT, DNA was pelleted by centrifugation for 5 min. at 2,000 *x g*. Protein-containing supernatant was precipitated with 1,125 μl ice-cold isopropyl alcohol and stored at -20°C o/n. Protein precipitates was pelleted by centrifugation at 12,000 *x g* for 10 min. Pellets were subsequently washed twice with 70% ethanol, subsequently rinsed with 100% ethanol before air dried at RT. Afterwards protein pellets were re-suspended in 50 mM ammonium-bicarbonate buffer (pH 8.0) containing 1% SDS, before reduced and alkylated with 5 mM DTT and 20 mM iodoacetamide, respectively. Subsequently the samples were rebuffered into 50 mM ammonium-bicarbonate buffer on filter columns (MWCO 10 kDa, Amicon, Millipore) and protein amount was determined by Pierce BCA protein assay kit. Protein digest and LC-MS measurement was performed as described in Hansmeier et al. [67]. Briefly, proteins were digested using trypsin gold (Promega, Madison) according manufacturers instruction before vacuum dried. Dried digested samples were resuspended in 0.1% formic acid and 3% acetonitrile and spiked with rabbit phosphorylase B (Waters Corporation, Milford, MA) for quantification before LC-MS analyses. Each sample was analyzed via a Waters NanoAcquity system coupled to a Waters Synapt G2 HDMS. A Waters NanoAcquity UPLC Symmetry C18 trap column (180 μm × 20 mm, dp: 5 μm) was used for desalting and focusing of peptides prior to their elution onto the Waters Acquity UPLC M-class HSS T3 analytical column (75 μm × 200 mm, dp: 1.8 μm) using a 120 min. gradient from 3% acetonitrile/0.1% formic acid to 45% acetonitrile/0.1% formic acid at a flow rate of 0.35 μl/min. Eluting peptides were analyzed in positive MS^E^ resolution mode with 1 s scan time. Collision energy was set to constant 4 eV for low energy scans and ramped between 18 and 42 eV for high energy scans. To ensure mass accuracy, leucine enkephaline was measured as lock mass every 30 s. The resulting spectra were processed with the ProteinLynx Global Server (PLGS) v. 3.02 with Identity (Waters) and searched against a protein sequence database build with the Uniprot *Salmonella* reference proteome (downloaded March 2015), supplemented with the sequences of the Waters PhosB standard for quantitation. Search parameter were set to mass tolerance 8 ppm, trypsin specificity, one missed cleavage, stable modification carbamidomethyl, variable modification methionine oxidation, false discovery rate 1%. To adjust for variances between injections, the concentration values for each chromatographic run were normalized against the total femtomole of protein quantified per analysis. To determine significant differential protein amounts in STM mutants compared to WT, we employed Student’s t-test and used the Benjamini−Hochberg method to adjust for multiple hypothesis testing.

### Quantification of ROS generation by RAW264.7 macrophages

Cultivation and infection of RAW264.7 macrophages was performed as above. For inhibition of NADPH oxidase, DPI (10 μM final concentration) was added to the cells with gentamicin from 1 h p.i. to the end of the experiment. At 8 h p.i., macrophages were recovered and pelleted by centrifugation (5 min. with 500 *x g* at RT). Supernatant was discarded and the cells resuspended in 200 μl fresh pre-warmed medium. Relative quantification of ROS in the host cells was performed as described [27]. Briefly, dihydrorhodamine (DHR) 123 (Chemodex; St. Gallen, Switzerland; final concentration 0.42 mM) was added to the tube lid. By short centrifugation, mixing of host cell suspension and DHR 123 solution was synchronized and cells were incubated for 20 min at 37°C. Cells were incubated on ice for 10 min, pelleted by centrifugation (5 min. at 500 *x g*, 4°C), supernatant was discarded, and cells fixed with 3% PFA in PBS for 15 min. at 4°C. Cells were recovered by centrifugation and resuspended in Attune Focusing fluid (Thermo Fisher Scientific) supplemented with BSA. Samples were directly subjected to flow cytometry on an Attune NxT cytometer and green fluorescence intensities per cell were measured.

### Quantification of intracellular proliferation

Macrophages were seeded into 24 well plates at 200,000 cells per well (TPP, Switzerland) one day before infection. Host cells were infected with STM o/n cultures with a MOI of 1, and infection was synchronized by centrifugation for 5 min. at 500 x *g* at RT. After 25 min. of infection, extracellular bacteria were killed by 100 μg x ml^-1^ gentamicin for 1 h, followed by 10 μg x ml^-1^ for the remaining period of time. For inhibition of NADPH oxidase, PAO or DPI (Sigma-Aldrich) at 0.5 and 5 μM, respectively, were added 1 h p.i. 1 and 8 h p.i. Host cells were washed thrice with PBS and lysed by incubation with 0.1% Triton-X-100 in PBS for 10 min. on a rocking platform. Lysates were diluted if required and plated on MH agar plates. Colony forming units (CFU) of inoculum, and of lysates at 1 and 8 h p.i. were counted and the intracellular replication rate (x-fold) was calculated as quotient between CFU (1 h) and CFU (8 h).

### RNA isolation from intracellular STM and qPCR

For RNA extraction and qPCR, cells were infected as described before and harvested at 10 h p.i. Bacteria were isolated with minor modification as previously described. After washing with PBS containing 0.05% SDS and 0.5% desoxycholate, bacteria were pelleted by centrifugation. Resulting pellets were resuspended in 1 ml PBS supplemented with 200 μl ice-cold stop solution (95% ethanol, 5% phenol, saturated with 0.1 M citrate-buffer, pH 4.3), before shock-frozen in liquid nitrogen. Afterwards, samples were thawed on ice and bacteria were pelleted by centrifugation at 6,344 *x g* and 4°C for 20 min.

RNA was prepared according to the ‘hot phenol’ method [68, 69]. Pellets of three experiments were pooled for each strain. In brief, cells were lysed using a lysis buffer (0.5 mg x ml^-1^ lysozyme (Sigma-Aldrich) in TE buffer pH 8.0 (Promega) with 2% SDS (Sigma-Aldrich)). Afterwards, samples were buffered with 3 M sodium acetate buffer (pH 5.2) (Life technologies), before RNA was extracted with Roti-Aqua phenol (Roth) at 64°C. After centrifugation (15.000 *x g*, 4°C, 20 min.), the watery-phase was loaded on heavy phase lock gel tubes (5PRIME GmBH, Hilden) supplemented with chloroform for further purification. Nucleic acids were precipitated twice with a 30:1 mixture of absolute ethanol and 3 M NaOAc (pH 5.2) with o/n incubation steps at -20°C. Pellets were washed with 75% ethanol, air-dried and solved in RNase-free water. Samples were treated with RNase-free DNase I (NEB), before RNA concentrations were determined using a nano-photometer (Implen). cDNA synthesis was performed with the RevertAid First strand cDNA synthesis kit (Thermo Fisher Scientific), using 1 μg RNA and random hexamer primers. qPCR was performed with the Maxima SYBR Green/Fluorescein qPCR Master Mix (Thermo Fisher Scientific) using the iCycler with MyiQ module (BioRad). Data were normalized to expression levels of a house-keeping gene (*gapA*) and calculated in consideration of primer efficiencies determined using serial dilutions of cDNA. The qPCR of *sseF* was used as negative control and internal confirmation of gene deletion. Student’s *t*-test was used for statistical analyses. Oligonucleotides used in this study are listed in S4 Table.

### Generation of reporter plasmids and flow cytometry analyses

Plasmid p4889 (P_EM7_::DsRed P_*uhpT*_::sfGFP) with constitutive expression of DsRed and regulated expression of sfGFP was used as basis vector for the generation of dual color reporter plasmids for flow cytometry analyses. The *uhpT* promoter was replaced by promoter fragments of *treA*, *msrA*, or *trxA* by Gibson assembly of PCR fragments generated by PCR using primers listed in S4 Table and the resulting dual color reporter plasmids are described in Table 2. For generation of a plasmid as negative control, a frame shift mutation was introduced in the 5’ region of sfGFP.

**Table 2:** Plasmids used in this study.

Designation	Relevant genotype	promoter position nt of STM chromosome	Source
p4889	P_EM7_::DsRed P_*uhpT*_::sfGFP	4,001,264…4,001,493 comp.	This study
p5074	P_EM7_::DsRed P_*treA*_::sfGFP	1,905,459…1,905,758	This study
p5084	P_EM7_::DsRed P_*msrA*_::sfGFP	4,658,671…4,658,970 comp.	This study
p5085	P_EM7_::DsRed P_*trxA*_::sfGFP	4,138,227…4,138,526	This study

RAW264.7 macrophages were infected with STM WT, Δ*sseF* or Δ*ssaV* strains harboring various reporter plasmids. Infected cells were lysed 12 h p.i. in order to release intracellular bacteria. After removal of host cell debris by centrifugation for 5 min. at 500 *x g*, bacteria were recovered from supernatant by centrifugation for 10 min. at 20,000 *x g*, fixed with 3% PFA in PBS, washed and recovered in PBS. Bacteria from the inoculum were directly harvested by centrifugation and fixed as described above (S2 Fig).

Flow cytometry was performed on an Attune NxT instrument (Thermo Fischer Scientific) at a flow rate of 25 μl x min.^-1^. At least 10,000 bacteria were gated by virtue of the constitutive DsRed fluorescence. The intensity of the sfGFP fluorescence per gated STM cell was recorded and x-medians for sfGFP intensities were calculated.

### Bioinformatics analyses

Proteins were classified according to Gene Ontology (GO) and COG classification schemes and mapped onto pathways using KEGG. Transport proteins were extracted from the data file by using queries in protein descriptions using keywords such as “transport”, “transferase” and “import”, followed by manual curation. Protein groups were visualized using STRING.

Proteome data sets are available at PeptideAtlas (http://www.peptideatlas.org/PASS/PASS01301).

## Supporting information

S1 Table Proteome data intracellular STM WT, Δ*sseF*, Δ*ssaV* isolated from RAW264.7 macrophages. (XLSX)

S2 Table Proteome data STM WT and Δ*sseF* cultured *in vitro* in PCN minimal medium. (XLSX)

S3 Table Comparison of proteome data for STM WT and Δ*sseF*, *in vitro* vs. *in vivo*. (XLSX)

S4 Table Oligonucleotides used in this study. (DOCX)

S1 Fig Quantification of ROS in RAW264.7 macrophages. RAW264.7 cells were infected with STM WT, Δ*sseF*, or Δ*ssaV* at a MOI of 1. Non-internalized bacteria were eliminated by gentamicin treatment. If indicated, DPI was added for inhibition of NADPH oxidase. RAW264.7 cells were recovered 8 h p.i., incubated with dihydrorhodamine 123, fixed and subjected to flow cytometry analysis. A) Relative comparison of ROS amounts in RAW264.7 cells infected with STM WT with or without DPI treatment (blue or light blue bars, respectively). Non-infected cells served as control and were set to 100%. B) Representative data of rhodamine 123 fluorescence intensities (BL1-H) in non-infected and infected RAW264.7 cells, with and without DPI treatment as depicted in (A). C) Relative comparison of ROS amounts in RAW264.7 macrophages infected with STM WT (= 100%), Δ*ssaV* or Δ*sseF*. D) Examples for rhodomine 123 fluorescence (BL1-H) in RAW264.7 macrophages infected with strains shown in (C). Means and standard deviation (A, C) represent data from at least three biological replicates. Statistical analysis was performed using Student’s *t*-test, *p* < 0.05 was considered as significantly different.
(TIF)

S2 Fig Dual fluorescence reporters for analyses of stress response of intracellular STM. A) For analyses of the expression of candidate genes on the level of single cell intracellular STM, mid copy number reporter plasmids were generated with DsRed expression under control of the constitutive EM7 promoter, and sfGFP expression under control of an *in vivo* differentially regulated promoter. B) STM strains harboring various reporters were used to infect RAW264.7 macrophages. Host cells were lysed 12 h after infection, cell debris were removed and released bacteria were fixed, recovered and subjected to flow cytometry analyses. C). Bacteria-sized particles were selected by FSC/SSC and DsRed-positive cells (YL-1) were gated. The sfGFP fluorescence intensity (BL-1) of the DsRed-positive population was recorded. D). Example of population analyses for various reporters in the background of STM WT (blue), Δ*sseF* (grey) and Δ*ssaV* (orange).
(TIF)

S3 Fig Intracellular replication of STM in RAW264.7 macrophages upon inhibition of ROS generation. RAW264.7 macrophages were infected with stationary cultures of STM WT, Δ*ssaV* and Δ*sseF* for 25 min. Non-internalized bacteria were killed by gentamicin treatment (100 μg x ml^-1^ for 1 h, 10 μg x ml^-1^ for the rest of the experiment). If indicated, PAO (0.5 μM) or DPI (5 μM) were added 1 h p.i. Infected cell were lysed at 1 and 8 h p.i., colony forming units (CFU) were determined, and intracellular replication was calculated (CFU 8 h/CFU 1 h). Depicted are means and standard deviations of one of three biological replicates with each three technical replicates. Statistical analysis was performed using Student’s *t*-test, *p* < 0.05 was considered as significantly different.
(TIF)

S4 Fig Levels of proteins for central carbon metabolism of *ssaV*-deficient STM. Detected proteins were mapped against the pathways annotated as involved in the central carbon metabolism of STM according to KEGG. The enzymes are presented in ovals and distinct colors indicate the abundance of the specific protein in STM Δ*ssaV* compared to STM WT. Statistical analysis was performed as indicated for Fig 3.
(TIF)

S5 Fig Levels of proteins for amino acid metabolism of *ssaV*-deficient STM. Detected proteins were mapped against the pathways annotated as involved in the central amino acid metabolism of STM according to KEGG. The enzymes are presented in ovals and distinct colors indicate the abundance of the specific protein in STM Δ*ssaV* compared to STM WT. Statistical analysis was performed as indicated for Fig 3.
(TIF)

S6 Fig qPCR confirms differential expression of genes for ABC transporter subunits and amino acid biosynthesis in STM Δ*sseF* compared to WT. Infection of RAW264.7 macrophages was performed as described in Materials and Methods. Isolated bacteria of several replicates were pooled, RNA extracted, subscribed to cDNA and used for qPCR experiments. Data were normalized to the expression levels of the house-keeping gene *gapA*. Statistical analysis was performed using Student’s *t*-test and significances are indicated as follows: ***, *p* < 0.001.
(TIF)

S7 Fig Levels of ABC transporter and PTS proteins of *ssaV*-deficient STM. Depicted are proteins detected in WT and Δ*ssaV*, which are components of ABC transporters (blue background) or PTS (pink background) according to KEGG. Subunits are presented in ovals and distinct colors indicate the abundance of a specific protein in STM Δ*ssaV* compared to STM WT. Statistical analysis was performed as indicated for Fig 3.
(TIF)

S8 Fig Active metabolic pathways of *Salmonella* WT in RAW264.7 macrophages at 12 h p.i. RAW264.7 macrophages were infected with *Salmonella* WT, cultured o/n in LB broth with aeration, with a MOI of 25. 12 h p.i. host cells were lysed and the bacteria isolated by differential centrifugation steps. Pooled bacterial pellets of several replicates were used for protein isolation and analyzed via LC-MS^E^. Detected proteins were mapped against metabolic pathways of STM using KEGG mapper [30]. Blue lines indicate detected enzymes, catalyzing the specific reactions.
(TIF)

S9 Fig Establishment of the infection protocol for proteomic analysis of intracellular STM. To gain the maximal amount of bacteria isolated from RAW264.7 macrophages, parameters of gentamicin protection assays (compare [36]) were varied: A) Intracellular replication assay using different MOIs. RAW264.7 macrophages were infected with STM with MOI of 1, 10 or 25. Cells were centrifuged for 5 min. at 500 *x g* and infection proceeded for 25 min. Cells were washed three times with PBS and extracellular bacteria were eliminated by gentamicin treatment (100 μg x ml^-1^ for 1 h, 10 μg x ml^-1^ for the remaining experiment). 2 h and 16 h p.i. cells were washed with PBS, lysed using 0.1% Triton X-100 and lysates were plated on MH agar plates. To determine the x-fold-replication rate, the quotient of the determined CFU x ml^-1^ at 2 h and 16 h p.i. was calculated. B) Phagocytosis assay with different infection times. As described in A, MOI of 25 was used for infection and phagocytosis was determined as quotient of the obtained CFU x ml^-1^ 2 h p.i. and the actual number of bacteria used for infection (inoculum). Infection times of 20 or 45 min. were compared. C) Phagocytosis assay with various relative centrifugal fields. The protocol was performed as described in B, with an infection time of 45 min. Centrifugal fields were varied between 500 and 1,500 *x g*. D) Intracellular replication assay with different time points of lysis. Infection of RAW264.7 macrophages occurred as described in C, using a centrifugal field of 1,250 *x g*. Cells were lysed 2 h and 8 to 16 h p.i. E) Intracellular replication assay using the established protocol. RAW264.7 macrophages were infected as described in D, lysis occurred 2 h and 12 h p.i, using 1% Triton X-100 in PBS. All experiments were performed in technical triplicates. Statistical analysis was performed using Student’s *t*-test and is indicated as follows: *, *p* < 0.05; **, *p* < 0.01; ***, *p* < 0.001; n.s., not significant.
(TIF)
