# Supplementary material for: Proteomics of intracellular Salmonella enterica reveals roles of Salmonella pathogenicity island 2 in metabolism and antioxidant defense
Source: PLoS Pathog. 2019 Apr 22;15(4):e1007741. doi: 10.1371/journal.ppat.1007741 (PMC6497321; doi:10.1371/journal.ppat.1007741)
Supplement: S4 Table — (DOCX) [file ppat.1007741.s004.docx]

Table S 4. Oligonucleotides used for in this study.

| Designation | Sequence 5’-3’ |
| --- | --- |
| for generation of gene deletions | |
| ssaV-Del13-for | TGAGGGAGTCAGGGCGCAACAGTGGCTCAGTGTATGCGCGATTCCGGGGATCCGTCGACC |
| ssaV-Del13-rev2 | TGTCCGCCAACTCCTCTTCGCTAAGGTCAATACTTTCTACTGTAGGCTGGAGCTGCTTCG |
| sseF-Del13-for | TGATACTCTTATTGCTTAAATAACAGAACGAAATATGAAAATTCCGGGGATCCGTCGACC |
| sseF-Del13-rev | TTGGGCTAACAGGTTTCATGGTTCTCCCCGAGATGTATGATGTAGGCTGGAGCTGCTTCG |
| for control of gene deletions | |
| ssaV-DelCheck-for | GGGCTTGCAATGAGTTGTTC |
| RT-ssaN-rev | TAATAACGCTTCGCCCACGG |
| sseF-DelCheck-for | TTGGCGAGAGAGGCTTTTC |
| sseF-DelCheck-Rev | ACCCGCTCTTTTCCTTGTTG |
| for qPCR | |
| artJ-qPCR-for | AGTAAGCAGGTCGCCTTTAG |
| artJ-qPCR-rev | ATCGCATTCTGGTAGCTGTC |
| metQ-qPCR-for | CTGGTTTCCGTTGGTAAGAC |
| metQ-qPCR-rev | ATATCCAGCGAGGTAGGAAG |
| glyA-qPCR-for | TGAAATCGCTGACAGCATCG |
| glyA-qPCR-rev | TTTCAGCGCTACCGCTTTAC |
| sseF-qPCR-for | AAGGCAGCAGAGGGATTATG |
| sseF-qPCR-rev | CACAAGAGCAACGCTATCAC |
| gapA-qPCR-for | TGGCCGTATCGGTCGCATTG |
| gapA-qPCR-rev | ATCGCGTTCAGCGGTAACAC |
| for Gibson assembly cloning | |
| Vf-p4889 | ATGCGCAAAGGCGAAGAACTGTTTACCGGTGTGGTGCCGA |
| Vr-p4889 | GGCCGGCATCACCGGCGCCACAGGTGCGGTTG |
| 1f-p4889-PtreA | CTGTGGCGCCGGTGATGCCGGCCTCCTGCTCTCTGCGCACCGCAGCCT |
| 1r-PtreA-p4889 | GTTCTTCGCCTTTGCGCATCAAGTTATCTCCTTAGCGGTGAGCCAAAGCCAACG |
| f-p4889-PmsrA | CTGTGGCGCCGGTGATGCCGGCCCTTCGACTTTCAGACGAACATTTGC |
| 1r-PmsrA-p4889 | GTTCTTCGCCTTTGCGCATGAGCTATTCTCCCGAAAGCGT |
| 1f-p4889-PtrxA | CTGTGGCGCCGGTGATGCCGGCCCCTGCCCTGCAACGTCACGACCC |
| 1r-PtrxA-p4889 | GTTCTTCGCCTTTGCGCATATATAACTCCACAGGAATAAGCCTGGCGTGTTGGTGTAGCATTA |
| Check primer | |
| Vf-p4507 | ACTTTTCTCCTCTTTCCCGGGTACCACTAG |
| PtreA-Check-Rev | TTATCTCCTTAGCGGTGAGCCAAAGCCAAC |
| PmsrA-Check-Rev | TTGGGTTAGCTTACTGTGCCTGAGCAGTTC |
| PtrxA-Check-Rev | TAACTCCACAGGAATAAGCCTGGCGTGTTG |
