# Supplementary figures and images for: Proteomics of intracellular Salmonella enterica reveals roles of Salmonella pathogenicity island 2 in metabolism and antioxidant defense
Source: PLoS Pathog. 2019 Apr 22;15(4):e1007741. doi: 10.1371/journal.ppat.1007741 (PMC6497321; doi:10.1371/journal.ppat.1007741)

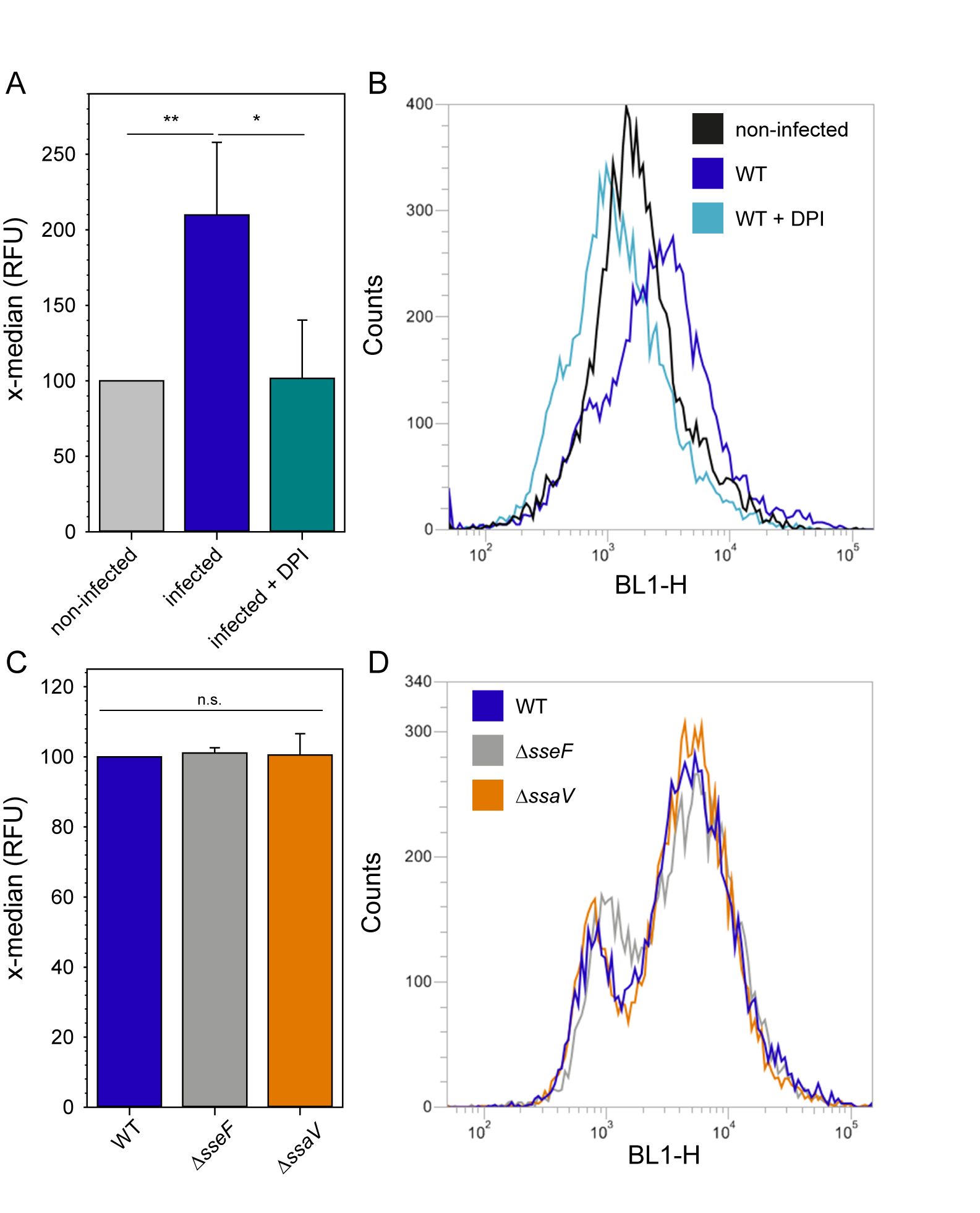

Supplement: S1 Fig — RAW264.7 cells were infected with STM WT, ΔsseF, or ΔssaV at a MOI of 1. Non-internalized bacteria were eliminated by gentamicin treatment. If indicated, DPI was added for inhibition of NADPH oxidase. RAW264.7 cells were recovered 8 h p.i., incubated with dihydrorhodamine 123, fixed and subjected to flow cytometry analysis. A) Relative comparison of ROS amounts in RAW264.7 cells infected with STM WT with or without DPI treatment (blue or light blue bars, respectively). Non-infected cells served as control and were set to 100%. B) Representative data of rhodamine 123 fluorescence intensities (BL1-H) in non-infected and infected RAW264.7 cells, with and without DPI treatment as depicted in (A). C) Relative comparison of ROS amounts in RAW264.7 macrophages infected with STM WT (= 100%), ΔssaV or ΔsseF. D) Examples for rhodomine 123 fluorescence (BL1-H) in RAW264.7 macrophages infected with strains shown in (C). Means and standard deviation (A, C) represent data from at least three biological replicates. Statistical analysis was performed using Student’s t-test, p < 0.05 was considered as significantly different. (TIF) [file ppat.1007741.s005.tif]

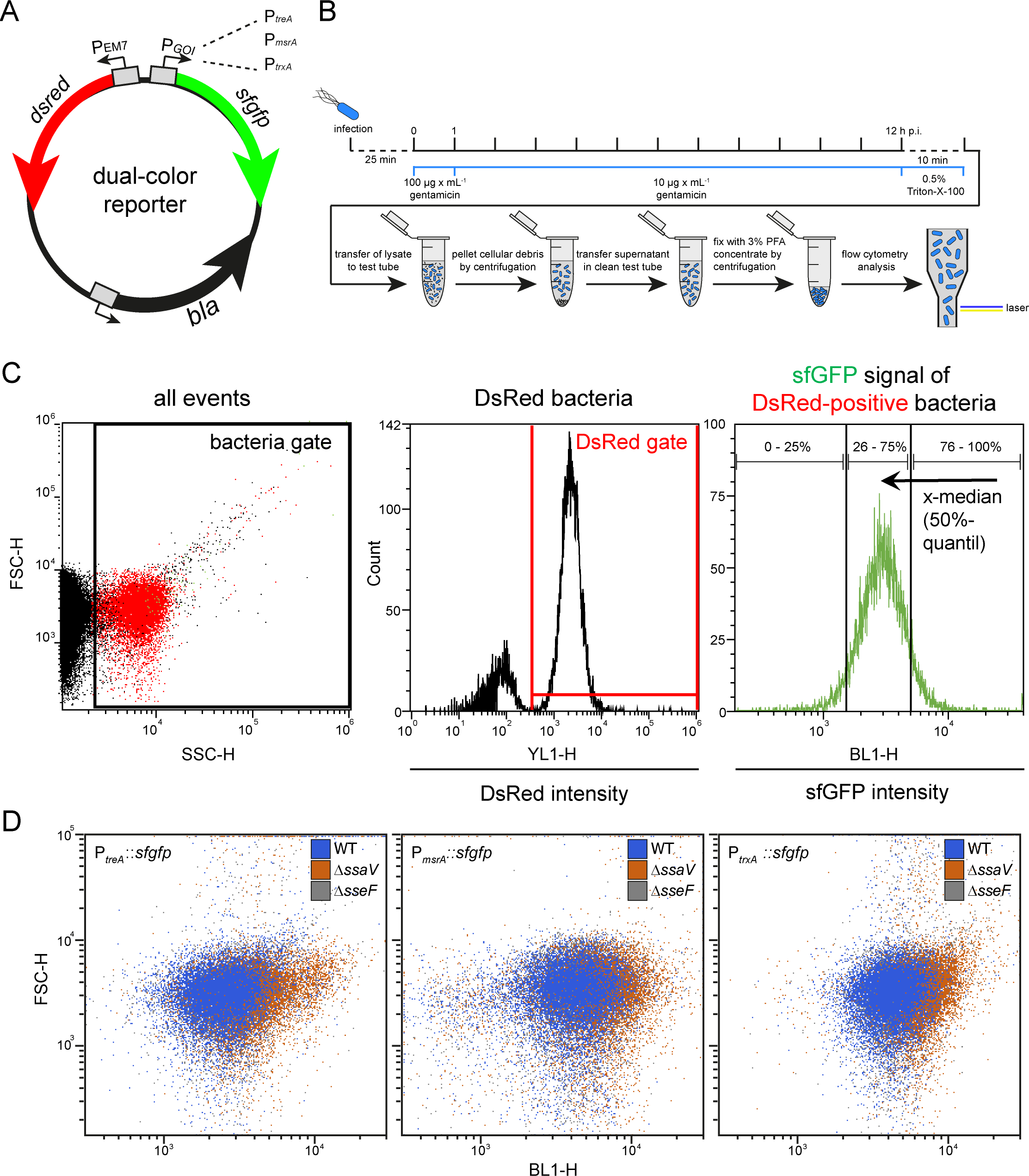

Supplement: S2 Fig — A) For analyses of the expression of candidate genes on the level of single cell intracellular STM, mid copy number reporter plasmids were generated with DsRed expression under control of the constitutive EM7 promoter, and sfGFP expression under control of an in vivo differentially regulated promoter. B) STM strains harboring various reporters were used to infect RAW264.7 macrophages. Host cells were lysed 12 h after infection, cell debris were removed and released bacteria were fixed, recovered and subjected to flow cytometry analyses. C). Bacteria-sized particles were selected by FSC/SSC and DsRed-positive cells (YL-1) were gated. The sfGFP fluorescence intensity (BL-1) of the DsRed-positive population was recorded. D). Example of population analyses for various reporters in the background of STM WT (blue), ΔsseF (grey) and ΔssaV (orange). (TIF) [file ppat.1007741.s006.tif]

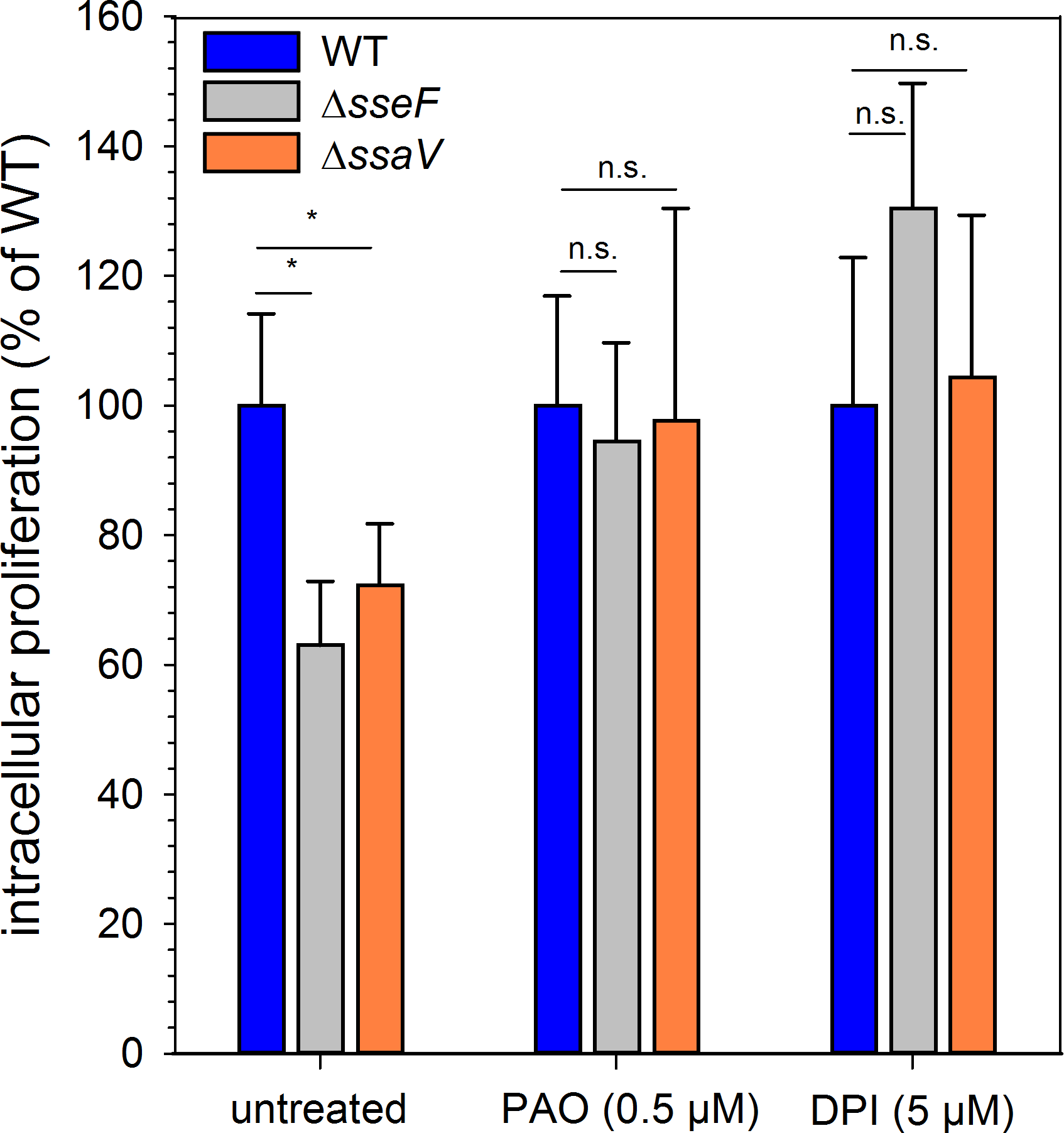

Supplement: S3 Fig — RAW264.7 macrophages were infected with stationary cultures of STM WT, ΔssaV and ΔsseF for 25 min. Non-internalized bacteria were killed by gentamicin treatment (100 μg x ml-1 for 1 h, 10 μg x ml-1 for the rest of the experiment). If indicated, PAO (0.5 μM) or DPI (5 μM) were added 1 h p.i. Infected cell were lysed at 1 and 8 h p.i., colony forming units (CFU) were determined, and intracellular replication was calculated (CFU 8 h/CFU 1 h). Depicted are means and standard deviations of one of three biological replicates with each three technical replicates. Statistical analysis was performed using Student’s t-test, p < 0.05 was considered as significantly different. (TIF) [file ppat.1007741.s007.tif]

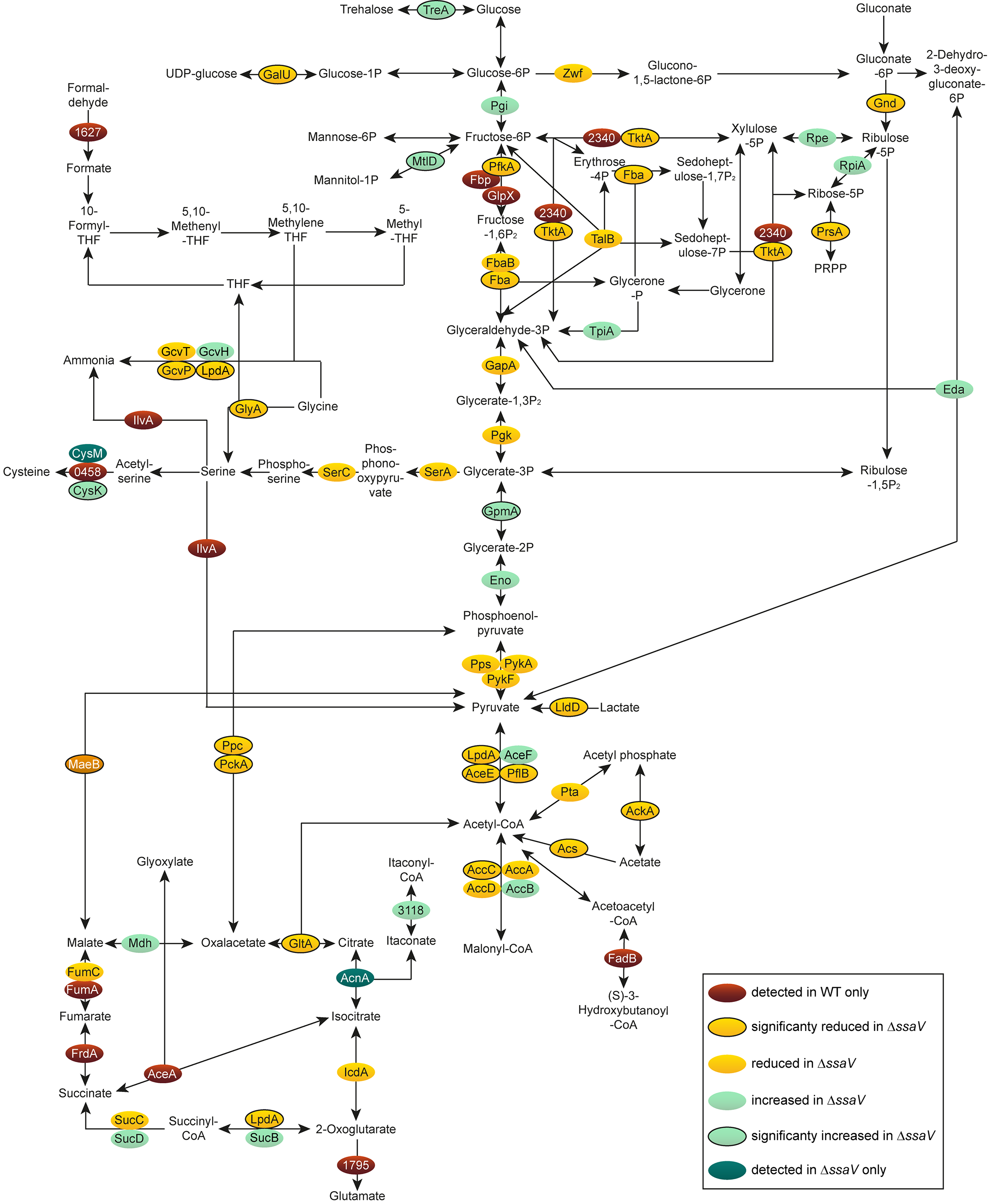

Supplement: S4 Fig — Detected proteins were mapped against the pathways annotated as involved in the central carbon metabolism of STM according to KEGG. The enzymes are presented in ovals and distinct colors indicate the abundance of the specific protein in STM ΔssaV compared to STM WT. Statistical analysis was performed as indicated for Fig 3. (TIF) [file ppat.1007741.s008.tif]

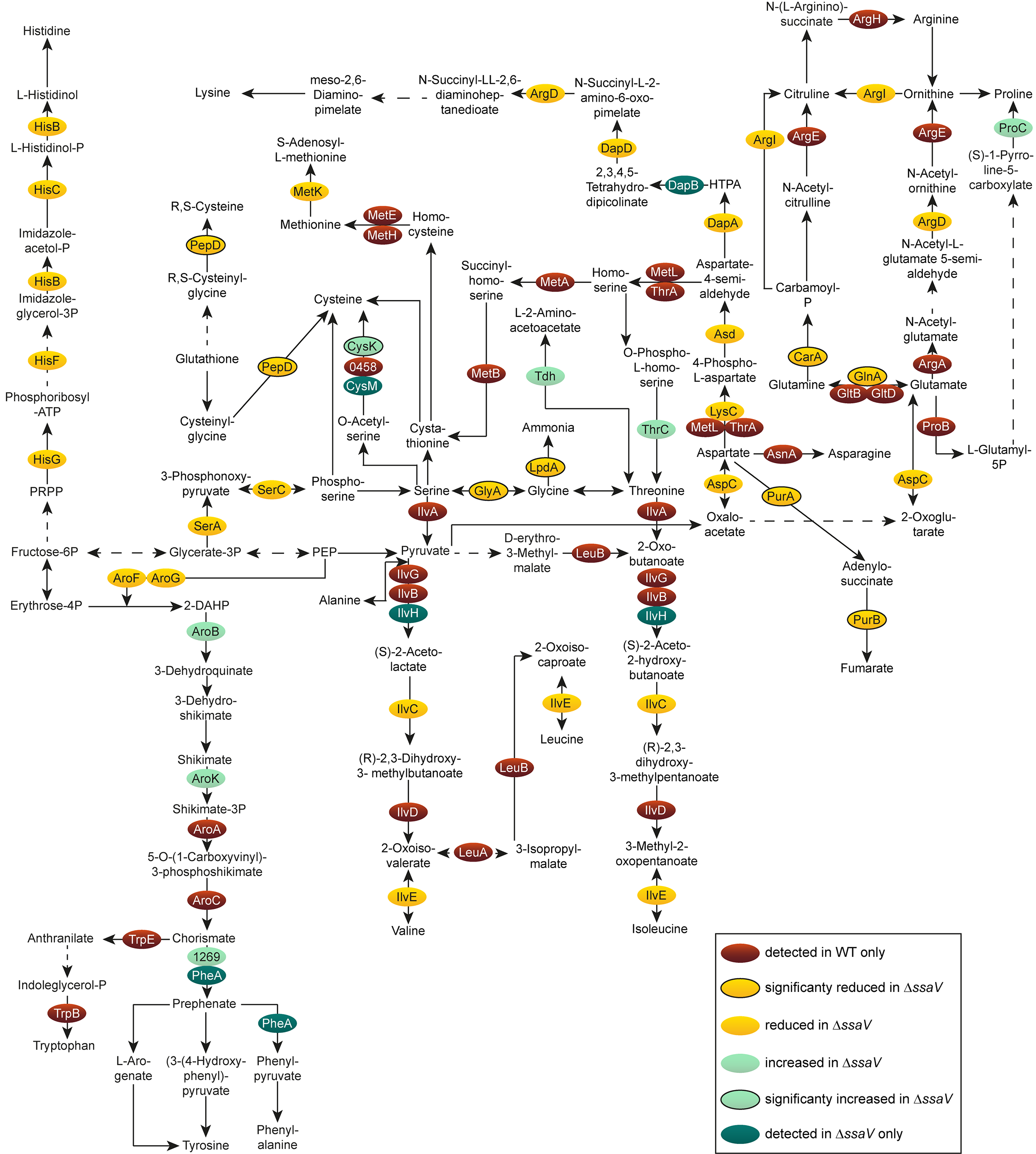

Supplement: S5 Fig — Detected proteins were mapped against the pathways annotated as involved in the central amino acid metabolism of STM according to KEGG. The enzymes are presented in ovals and distinct colors indicate the abundance of the specific protein in STM ΔssaV compared to STM WT. Statistical analysis was performed as indicated for Fig 3. (TIF) [file ppat.1007741.s009.tif]

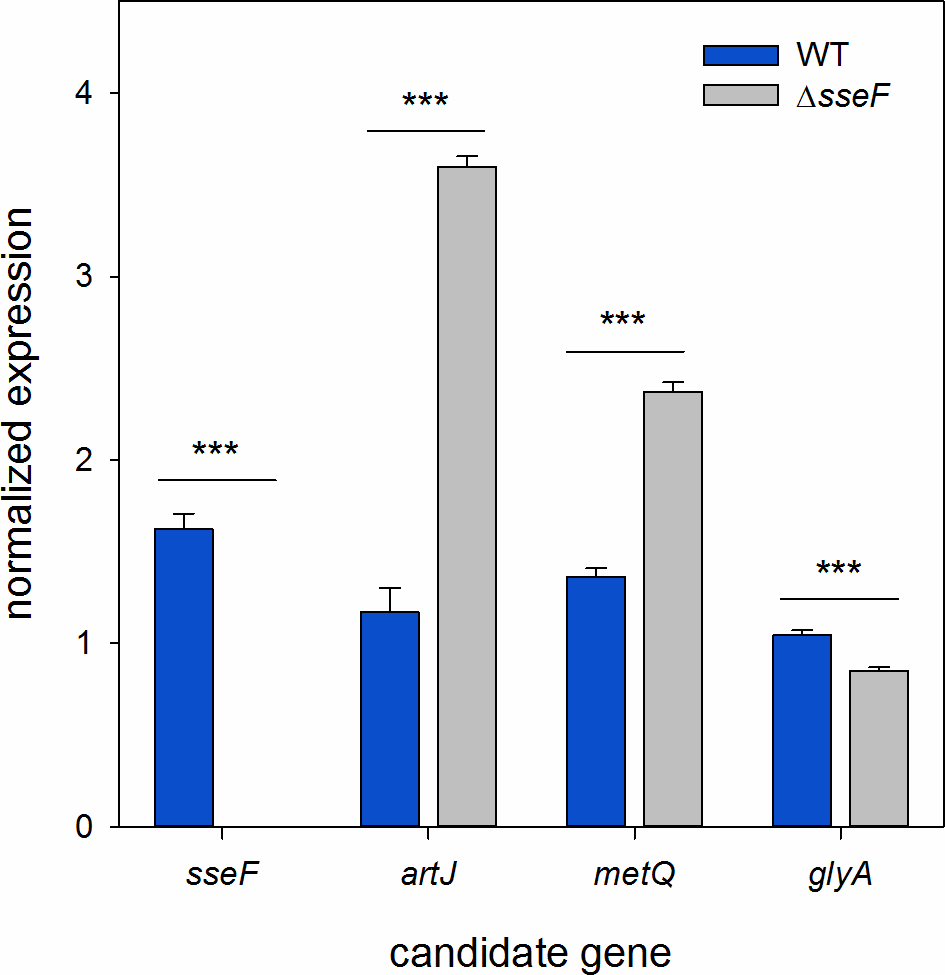

Supplement: S6 Fig — Infection of RAW264.7 macrophages was performed as described in Materials and Methods. Isolated bacteria of several replicates were pooled, RNA extracted, subscribed to cDNA and used for qPCR experiments. Data were normalized to the expression levels of the house-keeping gene gapA. Statistical analysis was performed using Student’s t-test and significances are indicated as follows: ***, p < 0.001. (TIF) [file ppat.1007741.s010.tif]

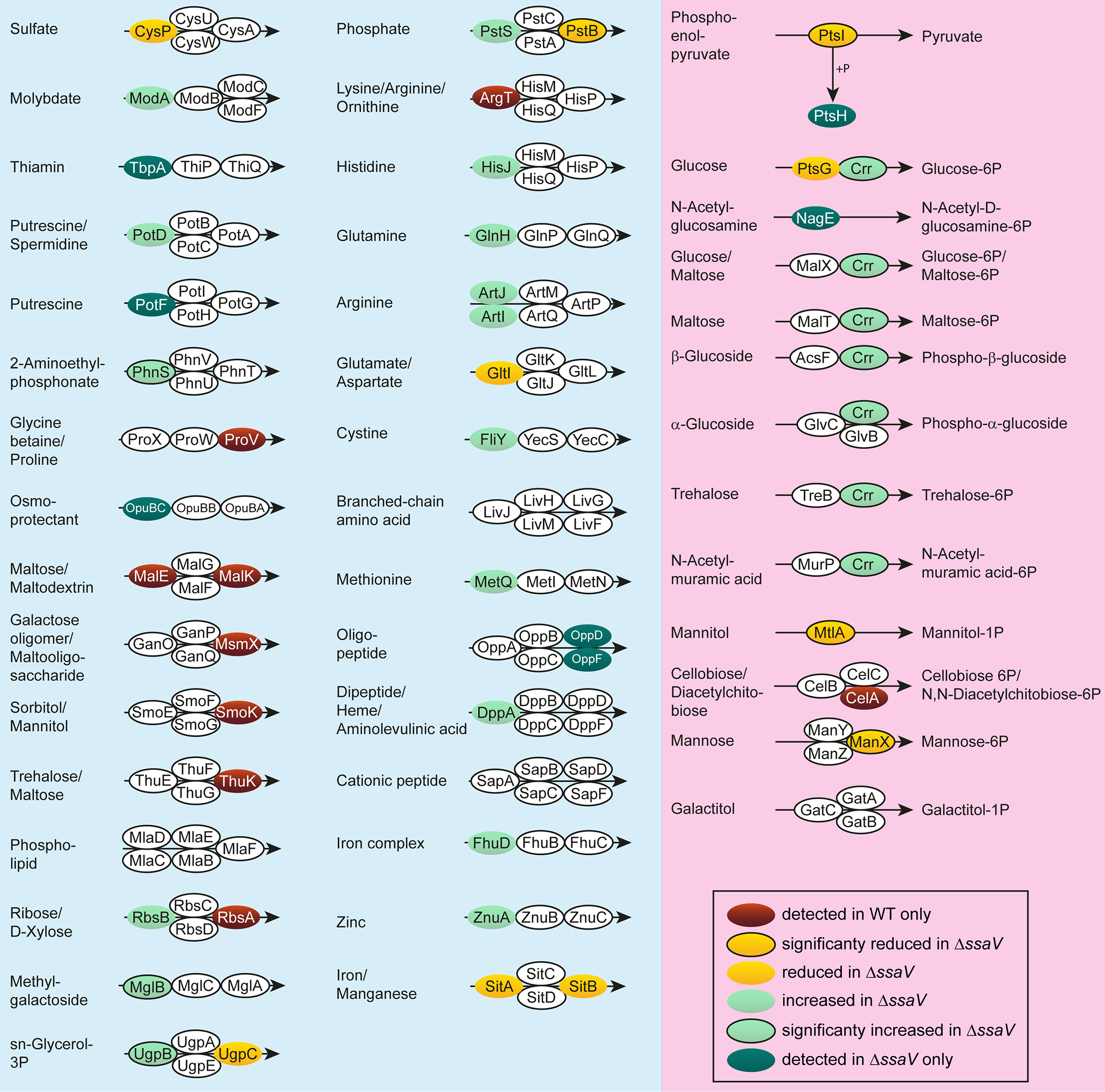

Supplement: S7 Fig — Depicted are proteins detected in WT and ΔssaV, which are components of ABC transporters (blue background) or PTS (pink background) according to KEGG. Subunits are presented in ovals and distinct colors indicate the abundance of a specific protein in STM ΔssaV compared to STM WT. Statistical analysis was performed as indicated for Fig 3. (TIF) [file ppat.1007741.s011.tif]

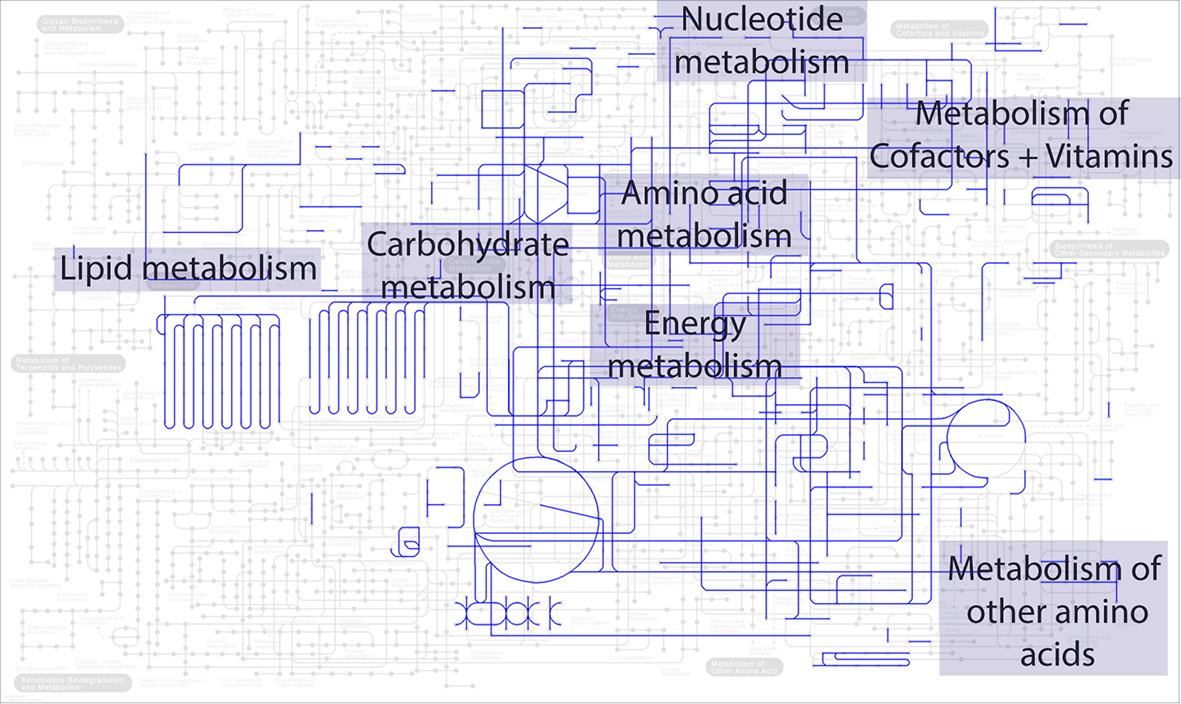

Supplement: S8 Fig — RAW264.7 macrophages were infected with Salmonella WT, cultured o/n in LB broth with aeration, with a MOI of 25. 12 h p.i. host cells were lysed and the bacteria isolated by differential centrifugation steps. Pooled bacterial pellets of several replicates were used for protein isolation and analyzed via LC-MSE. Detected proteins were mapped against metabolic pathways of STM using KEGG mapper [30]. Blue lines indicate detected enzymes, catalyzing the specific reactions. (TIF) [file ppat.1007741.s012.tif]

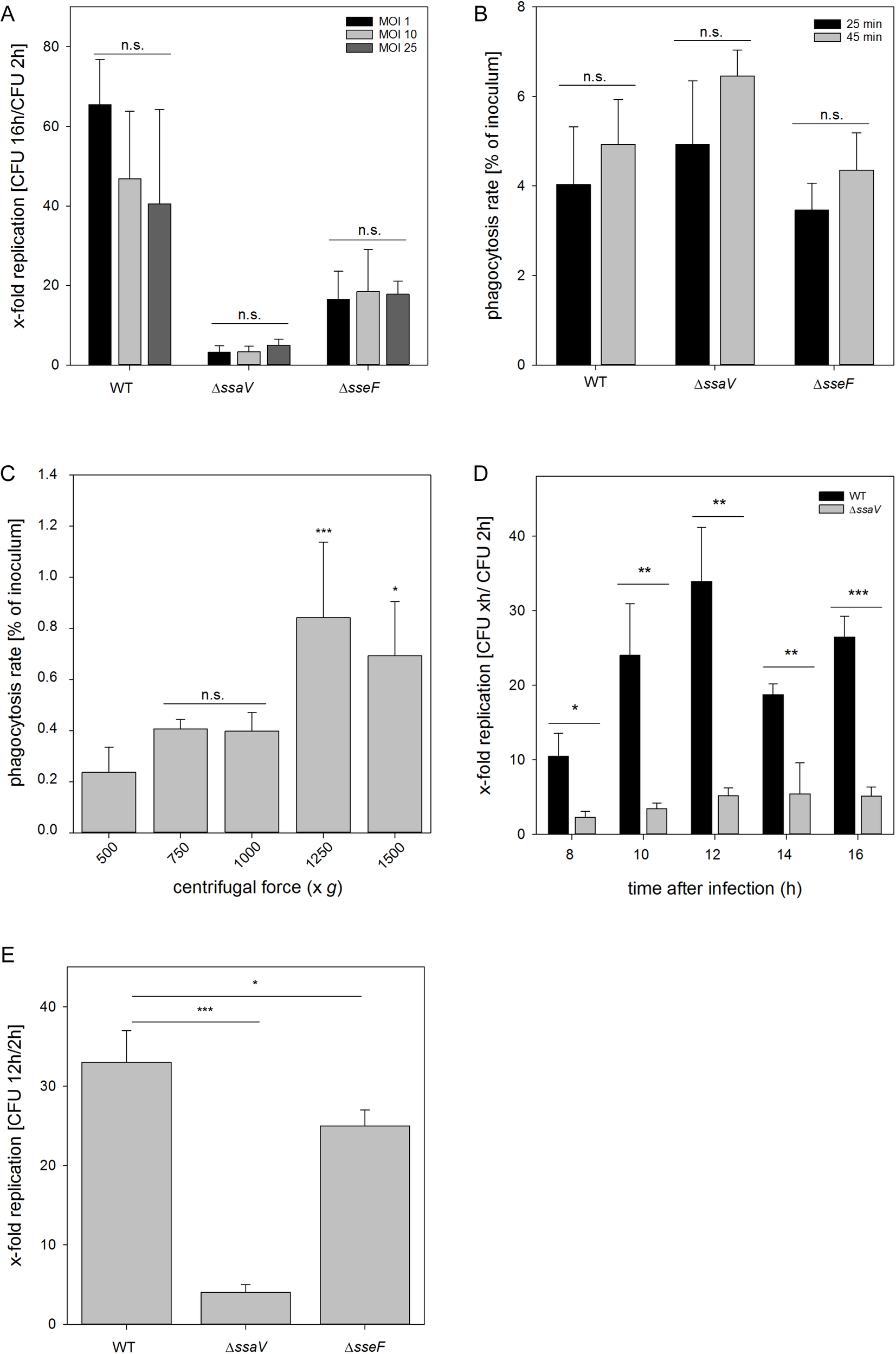

Supplement: S9 Fig — To gain the maximal amount of bacteria isolated from RAW264.7 macrophages, parameters of gentamicin protection assays (compare [36]) were varied: A) Intracellular replication assay using different MOIs. RAW264.7 macrophages were infected with STM with MOI of 1, 10 or 25. Cells were centrifuged for 5 min. at 500 x g and infection proceeded for 25 min. Cells were washed three times with PBS and extracellular bacteria were eliminated by gentamicin treatment (100 μg x ml-1 for 1 h, 10 μg x ml-1 for the remaining experiment). 2 h and 16 h p.i. cells were washed with PBS, lysed using 0.1% Triton X-100 and lysates were plated on MH agar plates. To determine the x-fold-replication rate, the quotient of the determined CFU x ml-1 at 2 h and 16 h p.i. was calculated. B) Phagocytosis assay with different infection times. As described in A, MOI of 25 was used for infection and phagocytosis was determined as quotient of the obtained CFU x ml-1 2 h p.i. and the actual number of bacteria used for infection (inoculum). Infection times of 20 or 45 min. were compared. C) Phagocytosis assay with various relative centrifugal fields. The protocol was performed as described in B, with an infection time of 45 min. Centrifugal fields were varied between 500 and 1,500 x g. D) Intracellular replication assay with different time points of lysis. Infection of RAW264.7 macrophages occurred as described in C, using a centrifugal field of 1,250 x g. Cells were lysed 2 h and 8 to 16 h p.i. E) Intracellular replication assay using the established protocol. RAW264.7 macrophages were infected as described in D, lysis occurred 2 h and 12 h p.i, using 1% Triton X-100 in PBS. All experiments were performed in technical triplicates. Statistical analysis was performed using Student’s t-test and is indicated as follows: *, p < 0.05; **, p < 0.01; ***, p < 0.001; n.s., not significant. (TIF) [file ppat.1007741.s013.tif]
